# Parental death and initiation of antidepressant treatment in surviving children and youth: a national register-based matched cohort study

**DOI:** 10.1016/j.eclinm.2023.102032

**Published:** 2023-06-08

**Authors:** Can Liu, Alessandra Grotta, Ayako Hiyoshi, Lisa Berg, Elizabeth Wall-Wieler, Pekka Martikainen, Ichiro Kawachi, Mikael Rostila

**Affiliations:** a Department of Public Health Sciences, Stockholm University, Stockholm, Sweden; b Centre for Health Equity Studies (CHESS), Stockholm University/Karolinska Institutet, Sweden; c Clinical Epidemiology Division, Department of Medicine, Karolinska Institutet, Solna, Stockholm, Sweden; d Clinical Epidemiology and Biostatistics, School of Medical Sciences, Örebro University, Örebro, Sweden; e Department of Epidemiology and Public Health, University College London, London, United Kingdom; f Manitoba Centre for Health Policy, University of Manitoba, Canada; g Population Research Unit, Faculty of Social Sciences, University of Helsinki, Finland; h Laboratory of Population Health, Max Planck Institute for Demographic Research, Germany; i Department of Social and Behavioral Sciences, Harvard School of Public Health, United States

**Keywords:** Parental death, Bereavement, Antidepressant, Depression, Anxiety, Children and youth

## Abstract

**Background:**

Population-based longitudinal studies on bereaved children and youth's mental health care use are scarce and few have assessed the role of surviving parents' mental health status.

**Methods:**

Using register data of individuals born in Sweden in 1992–1999, we performed a matched cohort study (n = 117,518) on the association between parental death and subsequent initiation of antidepressant treatment among individuals bereaved at ages 7–24 years. We used flexible parametric survival models to estimate the hazard ratios (HRs) over time after bereavement, adjusting for individual and parental factors. We further examined if the association varied by age at loss, sex, parental sociodemographic factors, cause of death, and the surviving parents' psychiatric care.

**Findings:**

The bereaved were more likely to initiate antidepressants treatment than the nonbereaved matched individuals during follow-up (incidence rate per 1000 person years 27.5 [26.5–28.5] vs. 18.2 [17.9–18.6]). The HRs peaked in the first year after bereavement and remained higher than the nonbereaved individuals until the end of the follow-up. The average HR over the 12 years of follow-up was 1.48 (95% confidence interval [1.39–1.58]) for father's death and 1.33 [1.22–1.46] for mother's death. The HRs were particularly high when the surviving parents received psychiatric care before bereavement (2.11 [1.89–2.56] for father's death; 2.14 [1.79–2.56] for mother's death) or treated for anxiety or depression after bereavement (1.80 [1.67–1.94]; 1.82 [1.59–2.07]).

**Interpretation:**

The risk of initiating antidepressant treatment was the highest in the first year after parental death and remained elevated over the next decade. The risk was particularly high among individuals with surviving parents affected by psychiatric morbidity.

**Funding:**

The 10.13039/501100004359Swedish Research Council.

**Research in context**

**Evidence before this study**

Parental death has been found to associate with depression and anxiety symptoms of children, especially in the first two years after the death. However, the investigation on the association between parental death and anxiety or depression is usually limited by the use of a small and non-representative sample, and it remains challenging to determine the longitudinal trajectory of depression and anxiety risk after parental death. Investigation of antidepressant prescriptions may shed some light on depression in bereaved children and youth, given their prevalent use of antidepressants. We searched PubMed and PsycINFO from database inception to 13 October, 2022, using the search terms ((parental death) OR (death of a parent) OR (bereavement in childhood) OR (bereaved child) OR (child griever) OR (grieving child)) AND (antidepressant) in English or other languages. We found three population-based studies, all from Denmark, showing an increased risk of antidepressant use following parental death. However, these studies did not delineate how the risk may change from the time of parental death to an extended period. Furthermore, we found no studies evaluating whether the surviving parents’ mental health may affect the association between parental death and antidepressant use among children and youth, although previous qualitative and quantitative studies using selected samples have suggested an important role played by the surviving parents.

**Added value of this study**

Using population-based multi-linked register data, this study showed that the association between parental death and antidepressant initiation changed over time. Among bereaved children and youth, the risk of antidepressant initiation peaked in the first year after parental death and remained elevated compared to nonbereaved individuals during the 12 years of follow-up. Children and youth's risk of initiating antidepressant treatment was especially high when their surviving parents suffered from psychiatric morbidities before or after bereavement.

**Implications of all the available evidence**

Parentally bereaved children and youth have a higher risk of mental health risk in both the short and long term compared to the nonbereaved. The association between parental death and antidepressant initiation is greater when the surviving parents have poor mental health, possibly because they could affect how their children's mental health problems are recognised and cared for. Bereavement support may focus more on the surviving parents and the interaction between the surviving parents and their children.

## Introduction

Parental death is one of the most traumatic experiences a child can have and is associated with an increased risk of depression and other adverse health and developmental outcomes later in life.^1^^,^^2^ It affects 3–4% of children in high-income countries,^1^^,^^3^ with the percentage possibly increasing during the COVID-19 pandemic.^4^

When a parent dies, the bereaved child undergoes considerable stress and may develop anxiety or depression symptoms, which may be treated with antidepressants.^5^ There have been a few population-based studies examining parentally bereaved children and youth's antidepressant use.^6^, ^7^, ^8^ However, none of them provided comprehensive analysis on how the risk of antidepressant initiation changed over time following parental death, although such information could be valuable for offering timely support for the bereaved child, as highlighted by a study examining 216 bereaved children over seven years.^9^ Furthermore, the negative impact of parental death may also exacerbate over long term, through accumulation of stressors subsequent to the death such as family socioeconomic difficulties and other changes in family life.^3^

Moreover, it remains unclear if the risk of antidepressant initiation differs by other individual and parental factors, such as cause of parental death, surviving parent's grief, age at experiencing parental death or parental education.^1^^,^^3^^,^^6^ Among the factors that may alleviate the impact of loss, a supportive environment and positive parenting provided by the surviving parent are vital for the child's adaptive grief response and achievement of developmental tasks.^5^^,^^9^, ^10^, ^11^, ^12^, ^13^ However, bereaved surviving parents are often challenged with parenting tasks^14^ and grief of losing the partner, as approximately 10% of bereaved adults have clinically recognised depression in the year after partner death.^15^^,^^16^ Nonetheless, there remains a paucity of evidence on how changing risk of antidepressant initiation varies by these characteristics.^3^^,^^10^

We aimed to evaluate the association between parental death and initiation of antidepressant treatment among children and youth bereaved at ages 7–24 years. We further evaluated how the association varies over time and by individual and familial characteristics, including psychiatric morbidity of their surviving parents.

## Methods

### Study design and population

**Fig. 1:**
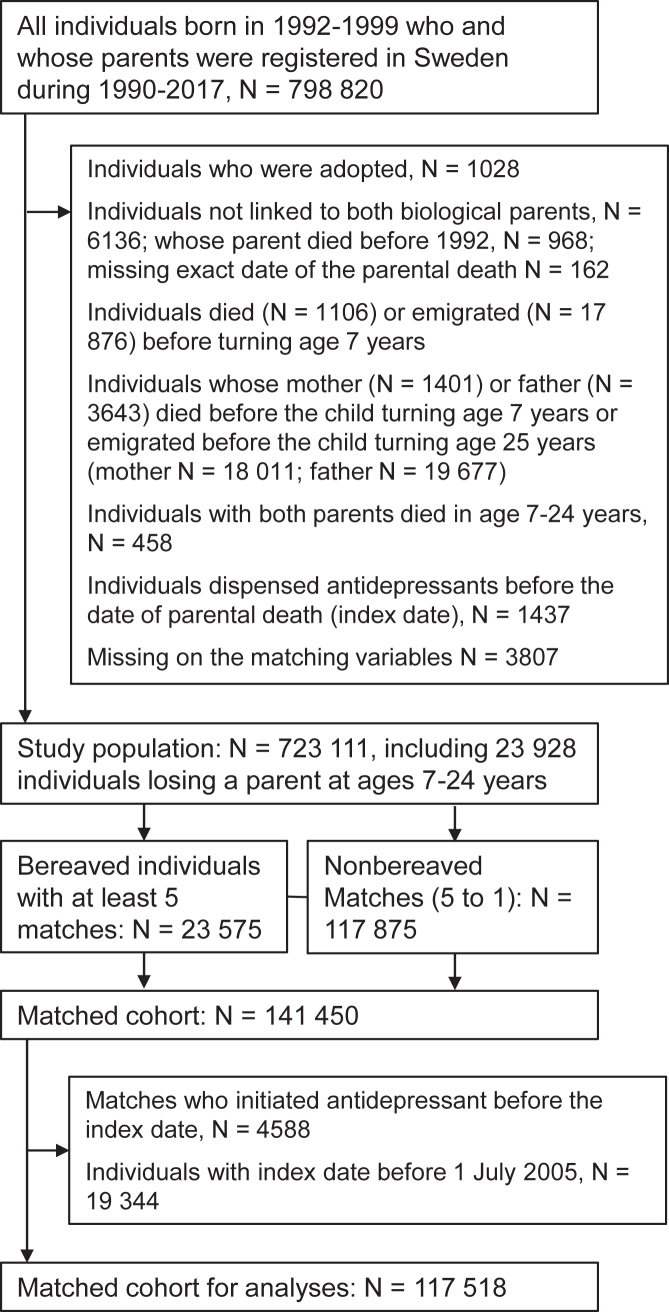
**Flowchart of the study population**. Index date:date of parental death, when follow-up started for the bereaved individuals and their matched nonbereaved individuals; Matching variables: sex, year of birth, maternal residence county in the year of birth, maternal age, and birth order.

We conducted a matched cohort study based on individuals born in 1992–1999 as recorded in the Swedish Medical Birth Register^17^ (N = 798,820) and linked with their biological parents through the Multigeneration Register.^18^ After several exclusions, the study population consisted of 723,111 individuals (Fig. 1).

Among the study population, 23,575 individuals lost a parent in ages 7–24 years old. To obtain a comparable group of nonbereaved individuals, we performed exposure density sampling^19^ to match the bereaved individuals to five individuals who have not been bereaved or matched until the month of the parental death. The bereaved and nonbereaved individuals were matched by sex, year of birth, birth order (1st, 2nd-3rd, 4th or higher), maternal age (in five-year categories) and maternal residence county in the year of childbirth. We defined an index date as the date of the parental death for both the bereaved and their nonbereaved matches. We excluded individuals who received antidepressant treatment before the index date. We further excluded all individuals with the index date before 1 July 2005 when the Prescribed Drug Register^20^ started. The final matched cohort consisted of 117,518 individuals, including 20,351 bereaved and 97,167 matched nonbereaved individuals (Fig. 1). Supplementary Figure S1 illustrates the measurements of variables and follow-up in calendar time and cohort's age in a diagram.

Data of the matched cohort members and their parents were collected from the following national registers: Longitudinal Integrated Database for Health Insurance and Labour Market Studies (LISA),^21^ the Total Population Register,^22^ the National Patient Register which includes both in- and out-patient care,^23^ the Prescribed Drug Register,^20^ and the Cause of Death Register.^24^ All linkages were performed using the Swedish unique personal identifier.^25^ All data were de-identified and cannot be used to identify any individual. The Swedish Ethical Review Authority approved this study (#2019–06175).

### Exposure

The date of parental death was collected from the Cause of Death Register. Individuals exposed to parental death were categorised as bereaved. When a parent of a nonbereaved matched individual died during the follow-up, the nonbereaved individual was censored and started to be followed up as bereaved, along with a new set of matched individuals. We also described the top ten causes of paternal and maternal death, respectively.

### Outcome

Mental health service is free for children in Sweden in an integrated primary care service system involving psychiatric and other health specialists. Antidepressants are prescribed primarily by psychiatric specialists for treating moderate to severe depression, anxiety disorders, and obsessive compulsory disorders in children.^26^^,^^27^ We defined the initiation of antidepressant treatment as the first recorded date of dispensing an antidepressant (Anatomical Therapeutic Chemical [ATC] code N06A).

### Confounders

The following variables were identified as potential confounding variables: sex (boy/girl), year of birth (continuous), maternal residence county at childbirth (categorical), maternal age at birth (in five-year categories), birth order (1st, 2nd-3rd, 4th or higher), maternal and paternal education in the year of childbirth (elementary, secondary, tertiary), parental foreign-born status (both Swedish-born, mother foreign-born, father foreign-born, both foreign-born), and deceased parent having psychiatric care, defined as having an in- or out-patient psychiatric care before death.

### Potential effect modifiers

Both individual and parental factors were collected and evaluated as potential effect modifiers. The individual factors included: age at the index date (7–12 years, 13–17 years, or 18–24 years) and sex (boy/girl). The parental factors included maternal and paternal education (elementary, secondary, or tertiary); parental foreign-born status (both Swedish born, mother foreign born, father foreign born, or both foreign born); cause of death (natural/unnatural); surviving parent's psychiatric care before bereavement, defined as having a diagnosis of psychiatric disorder from in- or out-patient psychiatric care prior to bereavement (yes/no); and surviving parent's anxiety or depression after bereavement (yes/no), defined as having a diagnosis of anxiety or depression from in- or out-patient care or filling at least one prescription for anxiolytics or antidepressants after bereavement (Supplementary Table S1 for the specific International Classification of Diseases Version 9 and 10 [ICD-9 and ICD-10] codes and Anatomical Therapeutic Chemical [ATC] codes).

### Statistical analyses

Bereaved and nonbereaved individuals were followed from the index date to the date of the first antidepressant dispensed, the date of death or emigration, or 31 December 2017, whichever came first. Nonbereaved matched individuals were also censored on the date of own parental death. Supplementary Figure S1 illustrates the follow-up period from 1 July 2005 to 31 December 2017 when the cohort member aged 7–25 years.

We plotted cumulative hazard curves for bereaved children and nonbereaved children using Nelson-Aalen estimator. We then used flexible parametric survival models comparing the bereaved children to the nonbereaved children to estimate average hazard ratios (HRs) and time-varying HRs with 95% confidence intervals (95% CIs), plotted over the follow-up time.^28^^,^^29^ The baseline rate and the time-varying HR were modelled with a restricted cubic splines with three degrees of freedom, which had the lowest Bayesian information criterion value (two knots at 33 and 67 percentiles of the uncensored event times). Both unadjusted and adjusted HRs for all the confounders were estimated and reported. All the analyses were conducted separately for maternal and paternal death. Cluster robust estimation was used to account for the matched clusters.

### Test of potential effect modification

To test potential effect modifiers that only vary within the bereaved group, we further categorised the bereaved group into subgroups defined by cause of death, surviving parent's psychiatric care before the death, and surviving parent's anxiety or depression after the death, to examine whether the association between parental death and antidepressant initiation differed by these potential effect modifiers. For example, the bereaved group was separated into two by cause of parental death (natural/unnatural), each compared with the nonbereaved reference group. Postestimation Wald test was used to compare between different categories of the bereaved groups.

To test potential effect modifiers that vary within both the bereaved and the nonbereaved group, we also performed stratified analyses by age at index date, sex of the child, maternal education, paternal education, and parental foreign-born status. Multiplicative interactions of exposure and potential effect modifiers were tested using likelihood ratio tests.

### Sensitivity analyses

First, to avoid the correlation of outcomes between siblings that contributes to an underestimation of the variance of the HR, we evaluated the association after having randomly selected one child from each family from the original matched cohort.

Second, as older youth may have moved away from the parents' household, we separately evaluated the effect modification of surviving parents’ psychiatric health among individuals bereaved before or after turning age 18.

Third, individuals who initiated antidepressant before the start of follow-up were excluded from analyses, as the research question required. However, imminent parental death may increase the risk of initiating antidepressant even before the index date. To explore the risk shortly before parental death and to test the robustness of the risk increase after the death, we performed a sensitivity analysis changing the index date as two years before the date of parental death. Matching and statistical analyses were performed in the same procedure as in the main analysis, except the splines for baseline rate and time-varying HR had two and four degrees of freedom respectively to fit the data. All analyses were performed using STATA® version MP 15.1 (StataCorp, College Station, TX). Two-sided P-values lower than 0.05 were considered to be significant.

## Role of funding

The funding source has no role in the writing of the manuscript or the decision to submission.

## Results

Out of the 723,117 individuals aged 7–24 years in the study population, 23,928 (3.3%) lost a parent (Supplementary Table S2). The most common cause of paternal death was acute myocardial infarction, followed by suicide. Atherosclerotic heart disease also ranked high (#4) in paternal death. In contrast, the dominant cause of maternal death was breast cancer. Suicide and acute myocardial infarction only ranked sixth and tenth among maternal death (Supplementary Table S3). Out of the 117,518 individuals in the matched cohort, 3126 bereaved and 10,479 nonbereaved individuals initiated antidepressant treatment. The bereaved were more likely to initiate the treatment at younger ages (p < 0.0001, Table 1). As expected, the distributions of sex, maternal age, and birth order were overlapping between the bereaved and matched nonbereaved individuals (Table 1).

**Table 1:** Characteristics of matched cohort (N = 117,518).

	All	No parental death	Parental death	P value
	N = 117,518	N = 97,167	N = 20,351	
	n (Col %)	n (Col %)	n (Col %)	
Antidepressant dispensed				<0.0001
Yes	13,605 (11.6)	10,479 (10.8)	3126 (15.4)	
No	103,913 (88.4)	86,688 (89.2)	17,225 (84.6)	
Age at first antidepressant dispensed (years)				<0.0001
7–12	61 (0.1)	46 (0)	15 (0.1)	
13–17	3025 (2.6)	2256 (2.3)	769 (3.8)	
18–24	10,230 (8.7)	7954 (8.2)	2276 (11.2)	
≥25 or no	104,202 (88.7)	86,911 (89.4)	17,291 (85.0)	
Age at index date (years)				<0.0001
7–12	22,154 (18.9)	18,469 (19)	3685 (18.1)	
13–17	50,292 (42.8)	41,718 (42.9)	8574 (42.1)	
18–24	45,072 (38.4)	36,980 (38.1)	8092 (39.8)	
Sex				0.082
Girl	55,583 (47.3)	45,845 (47.2)	9738 (47.9)	
Boy	61,935 (52.7)	51,322 (52.8)	10,613 (52.1)	
Maternal age at birth of the child				1.000
13–19	1930 (1.6)	1595 (1.6)	335 (1.6)	
20–24	14,658 (12.5)	12,106 (12.5)	2552 (12.5)	
25–29	34,412 (29.3)	28,458 (29.3)	5954 (29.3)	
30–34	38,169 (32.5)	31,574 (32.5)	6595 (32.4)	
35–39	23,343 (19.9)	19,296 (19.9)	4047 (19.9)	
40–44	5006 (4.3)	4138 (4.3)	868 (4.3)	
Birth order				0.965
1st born	38,823 (33.0)	32,108 (33.0)	6715 (33.0)	
2nd-3rd born	64,143 (54.6)	53,038 (54.6)	11,105 (54.6)	
4th or higher	14,552 (12.4)	12,021 (12.4)	2531 (12.4)	
Maternal education				<0.0001
Elementary	22,619 (19.2)	17,331 (17.8)	5288 (26)	
Secondary	44,092 (37.5)	36,247 (37.3)	7845 (38.5)	
Tertiary	50,807 (43.2)	43,589 (44.9)	7218 (35.5)	
Paternal education				<0.0001
Elementary	25,865 (22.0)	19,864 (20.4)	6001 (29.5)	
Secondary	46,184 (39.3)	38,191 (39.3)	7993 (39.3)	
Tertiary	45,469 (38.7)	39,112 (40.3)	6357 (31.2)	
Parental foreign-born status				<0.0001
Both Swedish born	93,497 (79.6)	78,037 (80.3)	15,460 (76)	
Mother foreign born	6138 (5.2)	4751 (4.9)	1387 (6.8)	
Father foreign born	6472 (5.5)	5172 (5.3)	1300 (6.4)	
Both foreign born	11,411 (9.7)	9207 (9.5)	2204 (10.8)	
Deceased parent having psychiatric care before the death				<0.0001
Yes	6914 (5.9)	373 (0.4)	6541 (32.1)	
No	110,604 (94.1)	96,794 (99.6)	13,810 (67.9)	
Surviving parent having psychiatric care before the death				<0.0001
Yes	3308 (2.8)	356 (0.4)	2952 (14.5)	
No	114,210 (97.2)	96,811 (99.6)	17,399 (85.5)	
Surviving parent having anxiety/depression after the death				<0.0001
Yes	42,725 (36.4)	35,052 (36.1)	7673 (37.7)	
No	74,793 (63.6)	62,115 (63.9)	12,678 (62.3)	

**Fig. 2:**
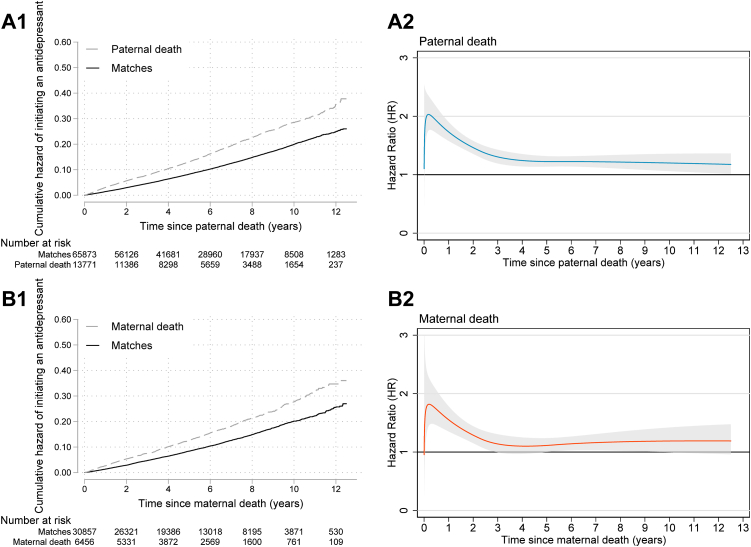
**Kaplan–Meier cumulative hazard functions and time-varying hazard ratio of initiating antidepressant treatment after parental death (A1 and A2: paternal death, B1 and B2: maternal death)**. Hazard ratio adjusted for sex, year of birth, birth order, maternal age and maternal residence county in the year of childbirth, maternal and paternal education, parental foreign-born status, and deceased parent having a psychiatric diagnosis before the death. Shaded areas indicate 95% confidence interval.

The median follow-up was 5.3 (interquartile range 2.9–8.3) years for the nonbereaved and 5.0 (2.7–8.0) years for the bereaved. The incidence rates were 18.2 [17.9–18.6] per 1000 person years for the nonbereaved and 27.5 [26.5–28.5] for the bereaved. The cumulative hazard of initiating an antidepressant was higher in the bereaved than the nonbereaved group by the end of 12.5 years of follow-up (Fig. 2 panel A1-B1). The average HR computed over the entire follow-up time was 1.48 [1.39–1.58] for father's death and 1.33 [1.22–1.46] for mother's death (Table 2). The time-varying HRs showed that the bereaved individuals had a twice higher risk than the nonbereaved individuals immediately after parental death. Subsequently, the risk gradually declined and an approximately 20% higher risk remained from the fourth year onward (Fig. 2 panel A2-B2). The pattern was not significantly different between individuals losing a mother and those losing a father (Supplementary Figure S2).

**Table 2:** Average hazard ratio of antidepressant initiation in bereaved individuals compared to the nonbereaved matches.

	Initiation of antidepressant treatment			
	Number of incidences	Incidence per 1000 person years [95% confidence interval]	Unadjusted hazard ratio [95% confidence interval]	Adjusted^a^ hazard ratio [95% confidence interval]
Paternal death				
Yes	2095	27.8 [26.7–29.0]	1.64 [1.56–1.73]	1.48 [1.39–1.58]
No	6831	18.2 [17.8–18.6]	1.00 [Reference]	1.00 [Reference]
Maternal death				
Yes	938	26.8 [25.1–28.6]	1.56 [1.44–1.68]	1.33 [1.22–1.46]
No	3182	18.3 [17.7–19.0]	1.00 [Reference]	1.00 [Reference]

a Adjusted for sex, year of birth, birth order, maternal age and maternal residence county in the year of childbirth, maternal and paternal education, parental foreign-born status, and deceased parent having a psychiatric diagnosis before the death.

**Fig. 3:**
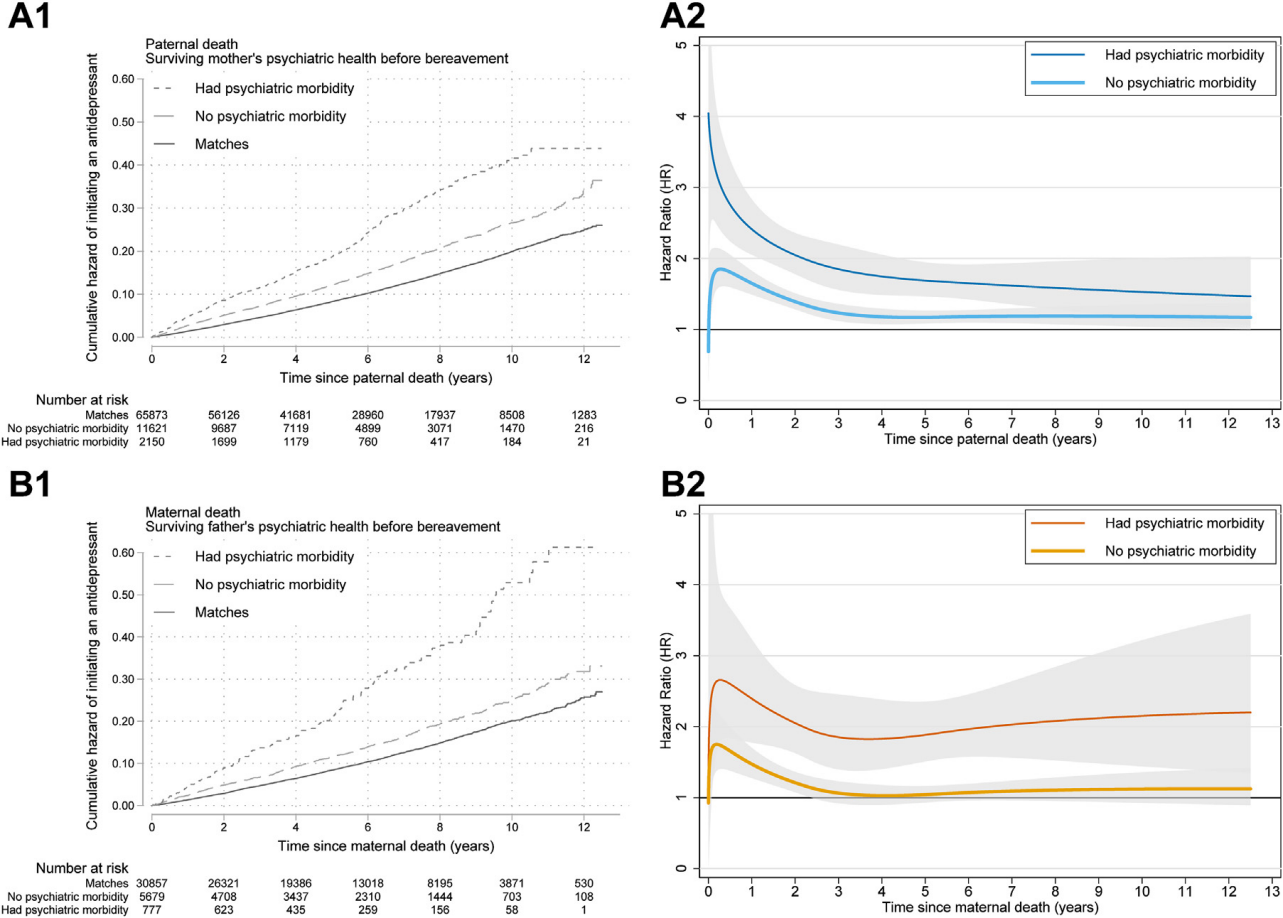
**Kaplan–Meier cumulative hazard functions and time-varying hazard ratio of initiating antidepressant treatment after parental death, by the surviving parent's psychiatric health before the death (A1 and A2: paternal death by the presence of surviving mother's psychiatric care before the death, B1 and B2: maternal death by the presence of surviving father's psychiatric care before the death)**. Hazard ratio adjusted for sex, year of birth, birth order, maternal age and maternal residence county in the year of childbirth, maternal and paternal education, parental foreign-born status, and deceased parent having a psychiatric diagnosis before the death. Shaded areas indicate 95% confidence interval.

**Fig. 4:**
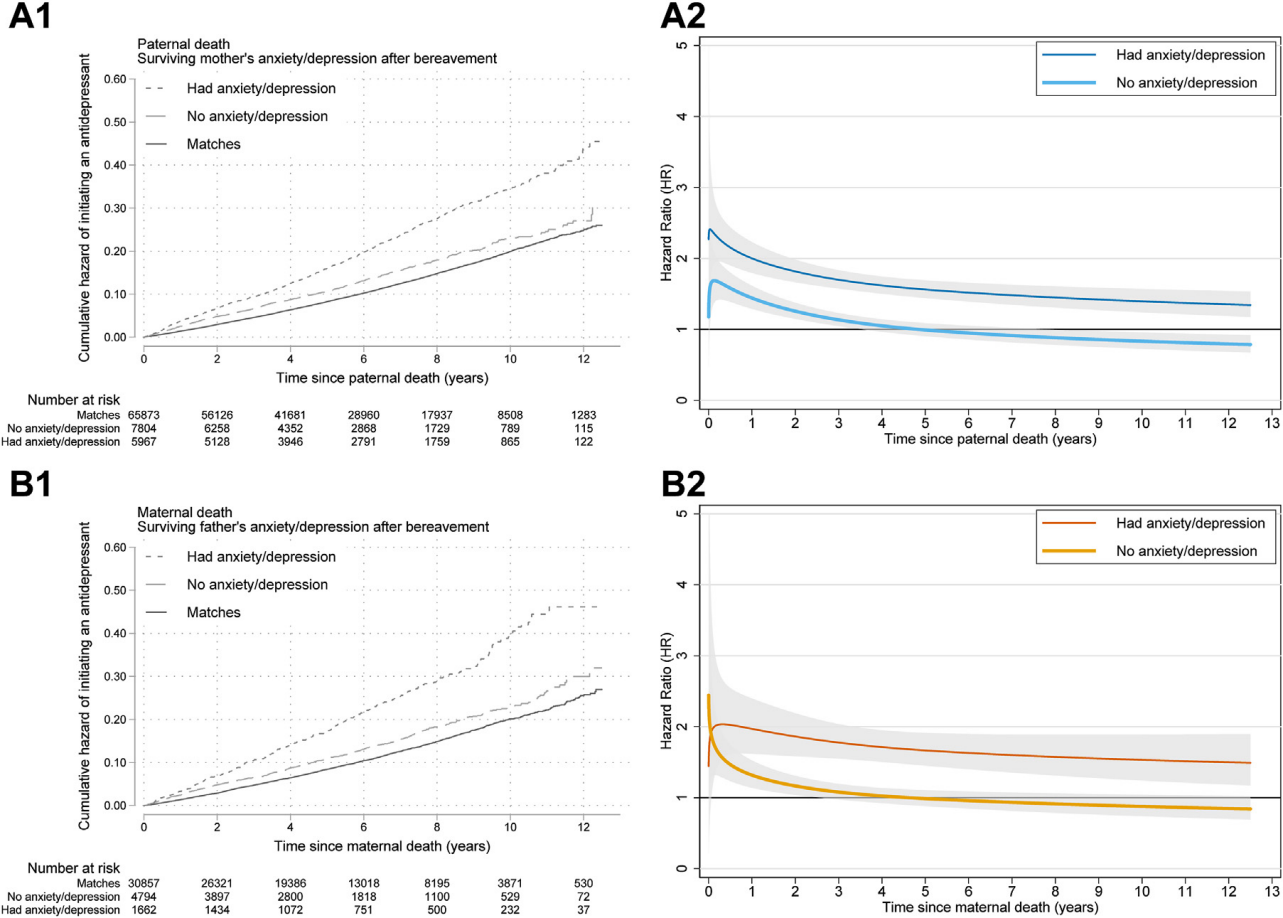
**Kaplan–Meier cumulative hazard functions and time-varying hazard ratio of initiating antidepressant treatment after parental death, by the surviving parent's anxiety/depression after the death (A1 and A2: paternal death by the presence of surviving mother's care for anxiety/depression after the death, B1 and B2: maternal death by the presence of surviving father's care for anxiety/depression after the death)**. Hazard ratio adjusted for sex, year of birth, birth order, maternal age and maternal residence county in the year of childbirth, maternal and paternal education, parental foreign-born status, and deceased parent having a psychiatric diagnosis before the death. Shaded areas indicate 95% confidence interval.

The overall HRs of bereaved children were even higher when surviving parents had psychiatric care before bereavement, compared with bereaved children whose parents did not have records of psychiatric care (2.14 [1.79–2.56] compared with 1.26 [1.15–1.38] after father's death; 2.11 [1.89–2.36] compared with 1.40 [1.31–1.49] after mother's death, Supplementary Table S4). The differences in HRs persisted throughout the follow-up time (Fig. 3 panel A2 and B2). Similar differences were observed by surviving parent's anxiety or depression after bereavement. Specifically, the bereaved children whose parents did not have depression or anxiety after bereavement had no elevated risk from the 4th year after bereavement and onward (Fig. 4 panel A2 and B2). The HRs did not differ by cause of the death (Supplementary Table S4), although mother's unnatural death was associated with elevated risk from the 4th year onward (Supplementary Figure S3 B2).

Younger age (7–17 years) at father's death may be associated with greater risk of antidepressant initiation (Table 3) than at ages 18–24 years, with the peak coming later and declined slower (Supplementary Figure S4b). In contrast, mother's death at ages 7–12 years showed no elevated risk throughout the follow-up (Table 3, Supplementary Figure S4a). The association between father's death and antidepressant initiation was stronger among boys than girls (Table 3, Supplementary Figure S5b), while risk of antidepressant initiation was generally lower among boys than girls (Supplementary Figure S5a). The association also differed by parental foreign-born status (Table 3), with slightly greater initial risk elevation but no increased risk in longer follow-up time among children with a foreign-born parent (Supplementary Figure S6a and 6b). The association did not differ by maternal or paternal education (Table 3, Supplementary Figure S7a and 7b). The association between mother's death and antidepressant initiation did not significantly vary by any of the potential effect modifiers.

**Table 3:** Average hazard ratios of antidepressant initiation in bereaved individuals compared to the nonbereaved matches, after paternal or maternal deaths separately and stratified by individual and parental sociodemographic characteristics.

	Initiation of antidepressant treatment			
	Paternal death		Maternal death	
	Adjusted^a^ hazard ratio [95% confidence interval]	P-value for interaction between parental death and stratifying variable	Adjusted^a^ hazard ratio [95% confidence interval]	P-value for interaction between parental death and stratifying variable
Age at parental death (years)		0.003		0.14
7–12	1.46 [1.29–1.67]		0.93 [0.76–1.13]	
13–17	1.46 [1.34–1.59]		1.39 [1.23–1.57]	
18–24	1.38 [1.23–1.55]		1.43 [1.21–1.68]	
Sex of the child		<0.0001		0.17
Girl	1.37 [1.27–1.49]		1.30 [1.15–1.45]	
Boy	1.68 [1.52–1.85]		1.41 [1.22–1.63]	
Maternal education		0.51		0.41
Elementary	1.45 [1.28–1.65]		1.57 [1.31–1.89]	
Secondary	1.45 [1.32–1.60]		1.28 [1.11–1.48]	
Tertiary	1.52 [1.38–1.69]		1.25 [1.07–1.46]	
Paternal education		0.73		0.66
Elementary	1.40 [1.25–1.58]		1.24 [1.03–1.50]	
Secondary	1.52 [1.38–1.67]		1.44 [1.25–1.66]	
Tertiary	1.49 [1.34–1.67]		1.31 [1.11–1.54]	
Parental foreign-born status		0.02		0.53
Both Swedish born	1.50 [1.40–1.61]		1.32 [1.19–1.46]	
Mother foreign born	1.43 [1.11–1.83]		1.51 [1.04–2.17]	
Father foreign born	1.36 [1.06–1.75]		1.14 [0.75–1.71]	
Both foreign born	1.38 [1.10–1.74]		1.69 [1.14–2.54]	

a The model is adjusted for sex, year of birth, birth order, maternal age and maternal residence county in the year of childbirth, maternal and paternal education, parental foreign-born status, and deceased parent having a psychiatric diagnosis before the death. The stratifying variable is excluded for adjustment.

Results of the first sensitivity analysis were consistent when randomly selecting one child in each family from the original matched cohort (and Supplementary Figure S8 and Supplementary Table S5). The second sensitivity analysis showed that effect modification by surviving parents’ psychiatric health presented in both children aged 7–17 years and youth aged 18–24 years (Supplementary Table S6). Last, when starting follow-up from two years before parental death, the HRs increased from before the death and peaked within one year after the date of parental death, as expected (Supplementary Figure S9).

## Discussion

Using longitudinal data of a national population, the study showed that the association between parental death and risk of initiating antidepressant treatment peaked in the first year after bereavement, gradually declined over subsequent two-three years, and remained elevated throughout the 12-years follow-up. The association was especially pronounced when the surviving parents had psychiatric morbidity before or after bereavement.

By modelling the changing risk of antidepressant initiation over 12 years after bereavement, this study found two important entry points for potential interventions to support parentally bereaved children and youth. First, the immediate increase of antidepressant initiation reflected an acute grief phase shortly after parental death. Meanwhile, receiving a prescription shortly after parental death may also suggest active care-seeking and response of the health care system. Second, the longer-term association that lasted throughout 12 years suggests that the bereaved children and youth need continuous support over time, especially in those whose surviving parents also suffer from depression and anxiety after bereavement. Adding to the previous study reporting an especially high risk of antidepressant use in two years after parental death,^8^ our findings suggest the risk elevation was peaking in the first year. Although our finding could not be used to conclude on depression symptoms, we observed a similar risk pattern over time as shown in a previous study on depression risk of 216 bereaved children.^9^ We also found that the pattern of the HR over time was similar after natural and unnatural parental death, which was not clear in previous studies limited to cancer or sudden parental death.^7^^,^^9^

To our knowledge, this is the first population-based study showing that the association between parental death and children and youth's antidepressant initiation is moderated by surviving parents' psychiatric health, as previously suggested by interview studies using smaller samples^3^^,^^13^ but has so far not been examined in population based studies. Although pre-existing genetic and environmental factors may explain the increased risk of psychiatric morbidity of both the surviving parents and their children, coping with loss is also a dynamic process that involves interactions between them.^30^ To better support the bereaved families, it is crucial to identify and act on amenable risk and protective factors, such as facilitating communication between the bereaved parent and child^31^ and promoting effective and positive parenting.^32^^,^^33^

Fathers' death had a stronger association with antidepressant initiation among children bereaved in 7–17 years, boys, and children having Swedish-born parents. Meanwhile, the association between mothers' death and the outcome did not differ by these factors. Such differences between paternal and maternal deaths may be partly attributable to the differences in cause of death, with paternal deaths more frequently being sudden deaths such as suicide. Mental health of risk children losing a parent to suicide may require further investigation, considering potential intergenerational transmissions of risks, the especially traumatic experience of the children, and the stigma around suicide.^34^^,^^35^ Parental education did not seem to modify the association between either fathers' or mothers' death and antidepressant initiation. These mixed findings by different characteristics of the children and their family may reflect potentially different psychiatric outcomes after parental death and differences in the children's care-seeking and access to psychiatric care.^1^^,^^6^ The current study could not differentiate the two. Thus, further studies may help to understand mechanisms generating the observed differences by population groups. Furthermore, our study did not explore the risk of long-lasting use of antidepressant, which may associate with parental bereavement.^8^ Future studies may need to explore antidepressant treatment duration and its combination with cognitive behavioural therapy, in addition to how the treatment continuation varies by the timing of treatment initiation and characteristics of the bereaved children and parents.

A strength of our study is that we used data of a national birth cohort with prospectively collected data. Through the Multigeneration Register, data of parents and children were linked and collected with minimal loss to follow-up. The register data also included a wide range of socioeconomic, demographic, and health variables with low missingness. Furthermore, register data guaranteed minimal measurement error on the exposure of parental deaths and outcome of filled prescriptions. Another strength and unique contribution of the study is using time since parental death as the time scale to continuously depict the changing risk over a long period of time, unlike previous studies which used age as time scale and parental death as time-dependent variable. By taking this analytical approach, we more clearly observed a rapid increase of HR after parental death than in previous studies.

Although the data has the strengths listed above, they did not provide all relevant information, especially the diagnoses information of children. Therefore, we cannot know the antidepressant treatment was associated with depression or anxiety related to the parental death. Furthermore, children may have non-pharmacological treatment instead of antidepressant. Thus, the finding may not be directly interpreted as the risk of depression or anxiety after parental death. Given the limited registered information, residual confounding, such as genetic factors transferred from parents to their children, likely existed even after matching and adjusting for a wide range of confounders measured at the start of follow-up. Although we captured all registered parental psychiatric morbidities before bereavement that was treated in specialised care, it is likely that some of the parents with psychiatric morbidity did not receive specialised care, resulting in an underestimation of psychiatric morbidity. It is also likely that these measurement errors will lead to residual confounding as well as an underestimation of effect modification by parental psychiatric morbidity. In a similar vein, children and youth who did not receive a prescription for antidepressant may not be considered as having no anxiety or depression or no long-term mental health risks. We also did not have information on when the child was informed about the disease or death of the parent, which may affect the association between parental death and child outcomes and should be explored in future studies.

Another potential limitation is a policy change to fully subsidise prescribed drugs for all children in Sweden from January 2016. Nonetheless, probably because the cost was already largely subsidised before the reform, there was little change in the constantly increasing trend of antidepressants consumption in children and youth.^36^^,^^37^ Thus, it is unlikely that the reform influenced the overall findings. Although our findings on antidepressant use after parental death may be more generalisable to countries with similar universal health care system, the implied anxiety or depression symptoms emerging in conjunction with parental death may be generalisable to other contexts. Finally, the findings may be generalisable to biological parent–child relationship only since we did not include adopted children due to the small number in this birth cohort.

We did not find strong effect modifications by parental education, which may affect health seeking behaviour.^6^ Nonetheless, future studies need to further explore the interplay between socioeconomic and psychosocial factors that changed after parental death and understand how they contributed to adverse child outcome in the long-term.

Parental death increased the risk of antidepressant initiation in childhood and youth. The risk peaked in the first year after parental death and remained elevated during the 12 years of follow-up. The risk was especially high in the presence of surviving parents’ psychiatric morbidity, suggesting that surviving parents may play an important role in the treatment for depression and anxiety of bereaved children and youth. Future research and supportive actions may treat bereaved family as a unit and pay more attention to mental health of both the surviving parents and their children, as well as the interaction between them.

## Contributors

CL, AG, AH, LB, and MR conceptualised the study and all authors contributed to refining the study concept and methods. CL prepared and performed the formal analysis and wrote the original draft of the manuscript. CL and AG assessed and verified the underlying data. MR supervised, acquired funding, obtained data, and did project administration. All authors participated in the interpretation of data, reviewed and edited the manuscript and had final responsibility for the decision to submit for publication.

## Data sharing statement

The source datasets supporting the conclusions of this article are available in the Swedish agencies’ repositories and can be made available to researchers after ethical approval. The raw data cannot be shared in a public repository based on data use agreement.

## Declaration of interests

All authors declare no conflict of interests.
