# Supplementary material for: Parental death and initiation of antidepressant treatment in surviving children and youth: a national register-based matched cohort study
Source: eClinicalMedicine. 2023 Jun 8;60:102032. doi: 10.1016/j.eclinm.2023.102032 (PMC10314171; doi:10.1016/j.eclinm.2023.102032)
Supplement: Supplementary Figures S1–S9 and Tables S1–S6 [file mmc1.docx]

Can Liu, Alessandra Grotta, Ayako Hiyoshi, Lisa Berg, Elizabeth Wall-Wieler, Pekka Martikainen, Ichiro Kawachi, Mikael Rostila

Supplementary for “Parental death and initiation of antidepressant treatment in surviving children and youth: a national register-based matched cohort study of individuals born in 1992-1999”

Table of Contents

[Supplementary Table 1. ICD-9 and ICD-10 Diagnoses 2](#_Toc134891566)

[Supplementary Table 2. Characteristic of the study population (N=723 117) 2](#_Toc134891567)

[Supplementary Table 3. Top 10 underlying causes of paternal and maternal deaths. 4](#_Toc134891568)

[Supplementary Table 4. Exposure breakdown analyses on the association between parental death and antidepressant initiation 4](#_Toc134891569)

[Supplementary Table 5. Hazard ratio of antidepressant initiation in bereaved individuals compared to the nonbereaved matches in the sample selecting only one child from each family 5](#_Toc134891570)

[Supplementary Table 6. Exposure breakdown analyses on the association between parental death and antidepressant initiation, among children bereaved at ages 7-17 years and at ages 18-24 years 5](#_Toc134891571)

[Supplementary Figure 1. Observation window for all individuals born from 1992 to 1999 6](#_Toc134891572)

[Supplementary Figure 2. Time-varying hazard ratio of initiating antidepressant treatment in maternally bereaved children compared to paternally bereaved children 7](#_Toc134891573)

[Supplementary Figure 3. Nelson-Aalen cumulative hazard functions and time-varying hazard ratio of initiating antidepressant treatment after parental death, by cause of parental death (A: paternal death by natural and unnatural cause of death, B: maternal death by natural and unnatural cause of death) 8](#_Toc134891574)

[Supplementary Figure 4a. Nelson-Aalen cumulative hazard functions of initiating antidepressant treatment after parental death, by age at parental death (A, C, and E: paternal death at age 7-12, 13-17, and 18-24 years, B, D, and F: maternal death at age 7-12, 13-17, and 18-24 years) 9](#_Toc134891575)

[Supplementary Figure 4b. Time-varying hazard ratio of initiating antidepressant treatment after parental death, by age at parental death (A, C, and E: paternal death at age 7-12, 13-17, and 18-24 years, B, D, and F: maternal death at age 7-12, 13-17, and 18-24 years) 10](#_Toc134891576)

[Supplementary Figure 5a. Nelson-Aalen cumulative hazard functions of initiating antidepressant treatment after parental death, by sex of the child (A and C: paternal death among boys and girls, B and D: maternal death among boys and girls) 11](#_Toc134891577)

[Supplementary Figure 5b. Time-varying hazard ratio of initiating antidepressant treatment after parental death, by sex of the child (A and C: paternal death among boys and girls, B and D: maternal death among boys and girls) 12](#_Toc134891578)

[Supplementary Figure 6a. Nelson-Aalen cumulative hazard functions of initiating antidepressant treatment after parental death, by parental foreign-born status (A and B: paternal and maternal death with both parents being Swedish-born, C and D: paternal and maternal death with a foreign-born mother and a Swedish-born father, E and F: paternal and maternal death with a foreign-born father and a Swedish-born mother, G and H: paternal and maternal death with both parents being foreign-born) 14](#_Toc134891579)

[Supplementary Figure 6b. Time-varying hazard ratio of initiating antidepressant treatment after parental death, by parental foreign-born status (A and B: paternal and maternal death with both parents being Swedish-born, C and D: paternal and maternal death with a foreign-born mother and a Swedish-born father, E and F: paternal and maternal death with a foreign-born father and a Swedish-born mother, G and H: paternal and maternal death with both parents being foreign-born) 16](#_Toc134891580)

[Supplementary Figure 7a. Nelson-Aalen cumulative hazard functions of initiating antidepressant treatment after parental death, by maternal or paternal education (A1 and A2: paternal death with maternal or paternal elementary education, C1 and C2: paternal death with maternal or paternal secondary education, E1 and E2: paternal death with maternal or paternal tertiary education. Correspondingly B1 and B2, D1 and D2, and F1 and F2 show maternal death with maternal or paternal elementary, secondary, and tertiary education) 17](#_Toc134891581)

[Supplementary Figure 7b. Time-varying hazard ratio of initiating antidepressant treatment after parental death, by maternal or paternal education (A1 and A2: paternal death with maternal or paternal elementary education, C1 and C2: paternal death with maternal or paternal secondary education, E1 and E2: paternal death with maternal or paternal tertiary education. Correspondingly B1 and B2, D1 and D2, and F1 and F2 show maternal death with maternal or paternal elementary, secondary, and tertiary education) 18](#_Toc134891582)

[Supplementary Figure 8. Nelson-Aalen cumulative hazard functions and time-varying hazard ratio of initiating antidepressant treatment after parental death, without sibling clustering (A1 and A2: paternal death, B1 and B2: maternal death) 19](#_Toc134891583)

[Supplementary Figure 9. Nelson-Aalen cumulative hazard functions and time-varying hazard ratio of initiating antidepressant treatment after parental death, with start of follow-up from two years before the actual date of death (A1 and A2: paternal death, B1 and B2: maternal death) 20](#_Toc134891584)

## Supplementary Table 1. ICD-9 and ICD-10 Diagnoses

| Natural cause of death | | ICD‐9 code: 000‐796; and ICD‐10 code: A00‐R99 | If the cause was not determined as unnatural cause, the disease cause of death was determined based on the "Main cause of death". |
| --- | --- | --- | --- |
| Unnatural cause of death | Accident | ICD‐9 code: 800‐999; and ICD‐10 code: V01‐Y98 | If the death was not suicide, the external cause was determined based on the "Main cause of death". |
|  | Suicide | ICD 9 code: E950–E959, E980–E989; ICD‐10 code: X60–X84, Y10–Y34 | Suicide was determined if any of the recorded "Main cause of death" or the "Contributing cause of death" was suicide. |
| Psychiatric care | | ICD-9 code:290-319; ICD-10 code: start with F | Measured as having main diagnosis for hospitalization |
| Anxiety | | ICD-9 code: 300.0, 300.01, 300.02, 300.23, 300.3, 300.8, 300.9; ICD-10 code: F40.1, F41.0, F41.1 F41.2, F41.3, F41.8, F41.9, F42.0, F42.1, F42.2, F42.8, F42.9, F48.9, F48.0 | Measured as having main diagnosis for hospitalization or specialized outpatient care |
| Depression | | ICD-9 code: 300.4; ICD-10 code: F32.0, F32.1, F32.2, F32.8, F32.9, F33.0, F33.2, F33.4, F33.8, F33.9 | Measured as having main diagnosis for the hospitalization or specialized outpatient care |
| Anxiolytics | | ATC code: N05B | Measured as filled prescription |
| Antidepressants | | ATC code: N06A | Measured as filled prescription |

## Supplementary Table 2. Characteristic of the study population (N=723 117)

|  | **All** | **No parental death** | **Parental death** |
| --- | --- | --- | --- |
|  | **N = 723 117** | **N=699 183** | **N=23 928** |
|  | **n (Col %)** | **n (Col %)** | **n (Col %)** |
| Antidepressant dispensation |  |  |  |
| Yes | 104 300 (14·4) | 100393 (14·4) | 3907 (16·3) |
| No | 618 811 (85·6) | 598790 (85·6) | 20021 (83·7) |
| Age at first antidepressant dispensation (years) |  |  |  |
| 7-12 | 2726 (0·4) | 2699 (0·4) | 27 (0·1) |
| 13-17 | 33 867 (4·7) | 32 898 (4·7) | 969 (4·0) |
| 18-24 | 66 409 (9·2) | 63 586 (9·1) | 2823 (11·8) |
| >=25 or no | 620 109 (85·8) | 600 000 (85·8) | 20 109 (84·0) |
| Age at first parental death (years) |  |  |  |
| 7-12 | N/A | N/A | 6941 (29·0) |
| 13-17 | N/A | N/A | 8743 (36·5) |
| 18-24 | N/A | N/A | 8244 (34·5) |
| Sex |  |  |  |
| Girl | 351 824 (48·7) | 340 360 (48·7) | 11 464 (47·9) |
| Boy | 371 287 (51·3) | 358 823 (51·3) | 12 464 (52·1) |
| Maternal age at birth of the child |  |  |  |
| 13-19 | 14 037 (1·9) | 13 627 (1·9) | 410 (1·7) |
| 20-24 | 128 312 (17·7) | 125 253 (17·9) | 3059 (12·8) |
| 25-29 | 270 574 (37·4) | 263 674 (37·7) | 6900 (28·8) |
| 30-34 | 211 844 (29·3) | 204 306 (29·2) | 7538 (31·5) |
| 35-39 | 83 029 (11·5) | 78 313 (11·2) | 4716 (19·7) |
| 40-44 | 14 757 (2·0) | 13 520 (1·9) | 1237 (5·2) |
| 45 or older | 558 (0·1) | 490 (0·1) | 68 (0·3) |
| Birth order |  |  |  |
| 1st born | 290 129 (40·1) | 282 268 (40·4) | 7861 (32·9) |
| 2nd-3rd born | 384 652 (53·2) | 371 722 (53·2) | 12 930 (54·0) |
| 4th or higher | 48 330 (6·7) | 45 193 (6·5) | 3137 (13·1) |
| Maternal education |  |  |  |
| Elementary | 128 486 (17·8) | 122 198 (17·5) | 6288 (26·3) |
| Secondary | 274 771 (38·0) | 265 530 (38·0) | 9241 (38·6) |
| Tertiary | 319 854 (44·2) | 311 455 (44·5) | 8399 (35·1) |
| Paternal education |  |  |  |
| Elementary | 145 591 (20·1) | 138 513 (19·8) | 7078 (29·6) |
| Secondary | 298 507 (41·3) | 289 109 (41·3) | 9398 (39·3) |
| Tertiary | 279 013 (38·6) | 271 561 (38·8) | 7452 (31·1) |
| Parental foreign-born status |  |  |  |
| Both Swedish born | 586 798 (81·1) | 568 617 (81·3) | 18 181 (76·0) |
| Mother foreign born | 33 637 (4·7) | 32 007 (4·6) | 1630 (6·8) |
| Father foreign born | 37 468 (5·2) | 35 941 (5·1) | 1527 (6·4) |
| Both foreign born | 65 208 (9·0) | 62 618 (9·0) | 2590 (10·8) |

## Supplementary Table 3. Top 10 underlying causes of paternal and maternal deaths.

| **Paternal death** | | | | **Maternal death** | | | |
| --- | --- | --- | --- | --- | --- | --- | --- |
| **Rank** | **Cause** | **ICD-10*** | **Percentage*** | **Rank** | **Cause** | **ICD-10*** | **Percentage*** |
| 1 | Acute myocardial infarction, unspecified | I219 | 7.8 | 1 | Malignant neoplasm of breast of unspecified site | C509 | 18.2 |
| 2 | Intentional self-harm by hanging, strangulation and suffocation | X70 | 5.1 | 2 | Malignant neoplasm of unspecified part of bronchus or lung | C349 | 5.6 |
| 3 | Malignant neoplasm of unspecified part of bronchus or lung | C349 | 4.7 | 3 | Malignant neoplasm of colon, unspecified | C189 | 3.1 |
| 4 | Atherosclerotic heart disease of native coronary artery | I251 | 3.3 | 4 | Malignant neoplasm of ovary | C56 | 3.0 |
| 5 | Malignant neoplasm of brain, unspecified | C719 | 3.0 | 5 | Malignant melanoma of skin, unspecified | C439 | 2.9 |
| 6 | Malignant neoplasm of pancreas, unspecified | C259 | 2.6 | 6 | Intentional self-harm by hanging, strangulation and suffocation | X70 | 2.9 |
| 7 | Malignant melanoma of skin, unspecified | C439 | 1.9 | 7 | Malignant neoplasm of brain, unspecified | C719 | 2.8 |
| 8 | Malignant neoplasm of colon, unspecified | C189 | 1.7 | 8 | Malignant neoplasm of brain, unspecified | C259 | 2.8 |
| 9 | Malignant neoplasm of prostate | C61 | 1.6 | 9 | Malignant neoplasm of cervix uteri, unspecified | C539 | 2.4 |
| 10 | Malignant neoplasm of rectum | C20 | 1.6 | 10 | Acute myocardial infarction, unspecified | I219 | 2.3 |

*Deaths of the same causes occurred before 1997 were coded in ICD-9 and were counted apart.

## Supplementary Table 4. Exposure breakdown analyses on the association between parental death and antidepressant initiation

|  | **Initiation of antidepressant treatment** | | | |
| --- | --- | --- | --- | --- |
|  | **Paternal death** | | **Maternal death** | |
|  | **Adjusted* hazard ratio [95% confidence interval]** | **P-value for difference between coefficients** | **Adjusted* hazard ratio [95% confidence interval]** | **P-value for difference between coefficients** |
| Cause of parental death |  | 0·15 |  | 0·74 |
| Natural | 1·46 [1·37-1·56] |  | 1·34 [1·22-1·47] |  |
| Unnatural | 1·58 [1·42-1·75] |  | 1·38 [1·15-1·66] |  |
| No death | 1·00 [Reference] |  | 1·00 [Reference] |  |
| Surviving parent having psychiatric morbidity before the death |  | <0·0001 |  | <0·0001 |
| Yes | 2·11 [1·89-2·36] |  | 2·14 [1·79-2·56] |  |
| No | 1·40 [1·31-1·49] |  | 1·26 [1·15-1·38] |  |
| No death | 1·00 [Reference] |  | 1·00 [Reference] |  |
| Surviving parent having anxiety/depression after the death |  | <0·0001 |  | <0·0001 |
| Yes | 1·80 [1·67-1·94] |  | 1·82 [1·59-2·07] |  |
| No | 1·22 [1·13-1·32] |  | 1·18 [1·06-1·30] |  |
| No death | 1·00 [Reference] |  | 1·00 [Reference] |  |

* Adjusted for sex, year of birth, birth order, maternal age and maternal residence county in the year of childbirth, maternal and paternal education, parental foreign-born status, and deceased parent having a psychiatric diagnosis before the death.

## Supplementary Table 5. Hazard ratio of antidepressant initiation in bereaved individuals compared to the nonbereaved matches in the sample selecting only one child from each family

|  | **Initiation of antidepressant treatment** | | | |
| --- | --- | --- | --- | --- |
|  | **Number of incidences** | **Incidence per 1000 person years** | **Unadjusted hazard ratio [95% confidence interval]** | **Adjusted* hazard ratio [95% confidence interval]** |
| Paternal death |  |  |  |  |
| Yes | 1598 | 28·8 [27·4-30·0] | **1·70 [1·60-1·80]** | **1·51 [1·41-1·62]** |
| No | 6499 | 18·3 [17·9-18·7] | 1·00 [Reference] | 1·00 [Reference] |
| Maternal death |  |  |  |  |
| Yes | 697 | 27·8 [25·8-29·9] | **1·58 [1·46-1·72]** | **1·36 [1·23-1·50]** |
| No | 3044 | 18·57 [17·8-19·1] | 1·00 [Reference] | 1·00 [Reference] |

* Adjusted for sex, year of birth, birth order, maternal age and maternal residence county in the year of childbirth, maternal and paternal education, parental foreign-born status, and deceased parent having a psychiatric diagnosis before the death.

## Supplementary Table 6. Exposure breakdown analyses on the association between parental death and antidepressant initiation, among children bereaved at ages 7-17 years and at ages 18-24 years

|  | **Initiation of antidepressant treatment** | | | |
| --- | --- | --- | --- | --- |
|  | **Paternal death** | | **Maternal death** | |
|  | **Adjusted* hazard ratio [95% confidence interval]** | **P-value for difference between coefficients** | **Adjusted* hazard ratio [95% confidence interval]** | **P-value for difference between coefficients** |
| **Bereaved child aged 7-17 years** | | | | |
| Surviving parent having psychiatric morbidity before the death |  | <0·0001 |  | <0·0001 |
| Yes | **2·10 [1·84-2·39]** |  | **2·08 [1·69-2·56]** |  |
| No | **1·39 [1·29-1·50]** |  | **1·18 [1·05-1·31]** |  |
| No death | 1·00 [Reference] |  | 1·00 [Reference] |  |
| Surviving parent having anxiety/depression after the death |  | <0·0001 |  | <0·0001 |
| Yes | **1·83 [1·69-1·99]** |  | **1·74 [1·50-2·03]** |  |
| No | **1·15 [1·05-1·27]** |  | 1·08 [0·96-1·22] |  |
| No death | 1·00 [Reference] |  | 1·00 [Reference] |  |
| **Bereaved youth aged 18-24 years** | | | | |
| Surviving parent having psychiatric morbidity before the death |  | 0·0009 |  | 0·004 |
| Yes | **1·87 [1·53-2·30]** |  | **2·18 [1·57-3·02]** |  |
| No | **1·32 [1·17-1·49]** |  | **1·36 [1·15-1·61]** |  |
| No death | 1·00 [Reference] |  | 1·00 [Reference] |  |
| Surviving parent having anxiety/depression after the death |  | <0·0001 |  | <0·0001 |
| Yes | **1·77 [1·53-2·05]** |  | **2·14 [1·67-2·73]** |  |
| No | **1·17 [1·02-1·34]** |  | **1·26 [1·05-1·51]** |  |
| No death | 1·00 [Reference] |  | 1·00 [Reference] |  |

**
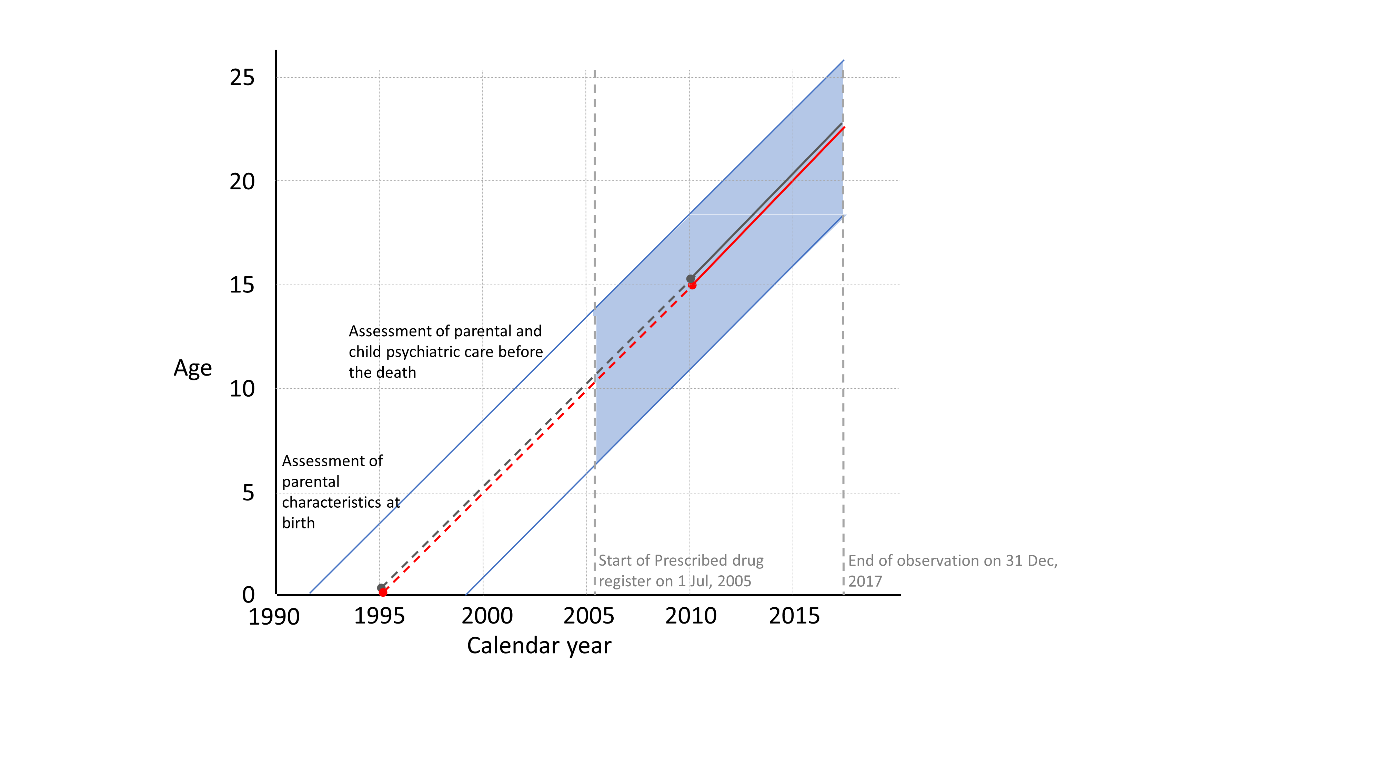
**

## Supplementary Figure 1. Observation window for all individuals born from 1992 to 1999

The blue area represents the period when parental death and antidepressant dispensation are observed given the available data. The solid red and dark grey lines represent the follow-up of a child and his/her five matches (all represented in one), starting at the index date (i.e. date of parental death for the bereaved child). The dashed lines represent the observation of the child and his/her matches and their parents before the death. The child and his/her matches were aged 15 years in 2010.


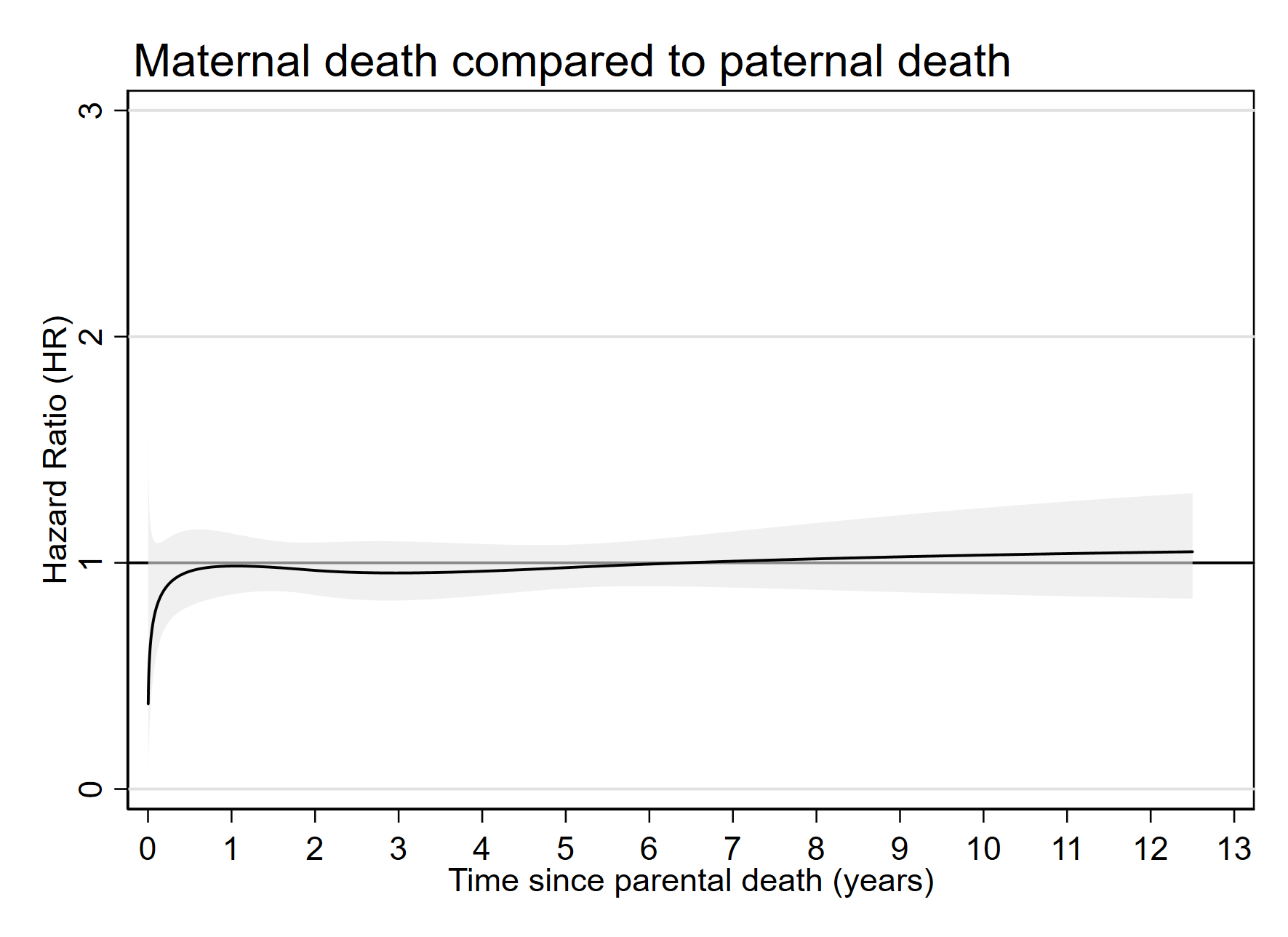


## Supplementary Figure 2. Time-varying hazard ratio of initiating antidepressant treatment in maternally bereaved children compared to paternally bereaved children

Hazard ratio adjusted for sex, year of birth, birth order, maternal age and maternal residence county in the year of childbirth, maternal and paternal education, parental foreign-born status, and deceased parent having a psychiatric diagnosis before the death. Shaded areas indicate 95% confidence interval.

**
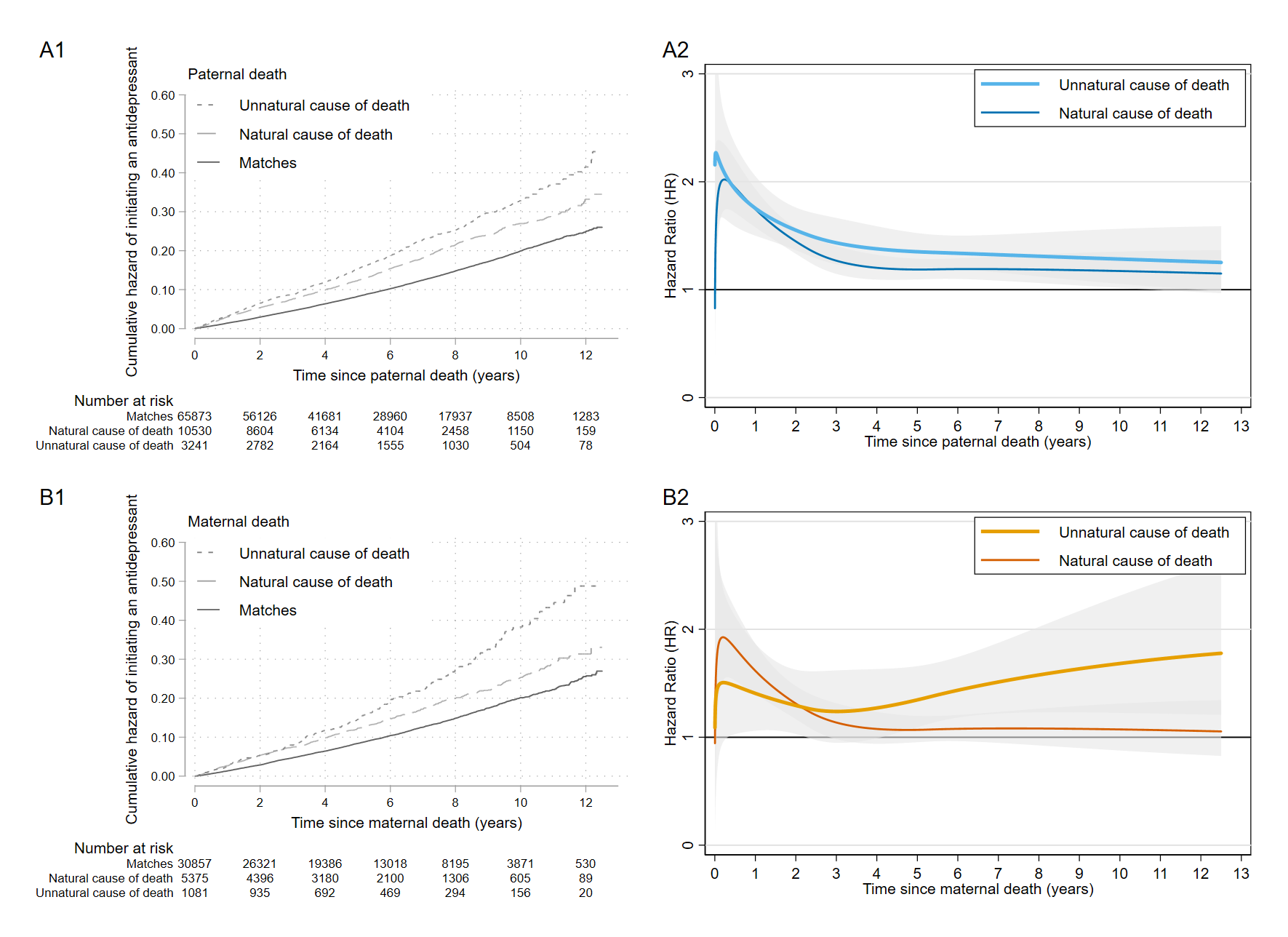
**

## Supplementary Figure 3. Nelson-Aalen cumulative hazard functions and time-varying hazard ratio of initiating antidepressant treatment after parental death, by cause of parental death (A: paternal death by natural and unnatural cause of death, B: maternal death by natural and unnatural cause of death)

Hazard ratio adjusted for sex, year of birth, birth order, maternal age and maternal residence county in the year of childbirth, maternal and paternal education, parental foreign-born status, and deceased parent having a psychiatric diagnosis before the death. Shaded areas indicate 95% confidence interval.


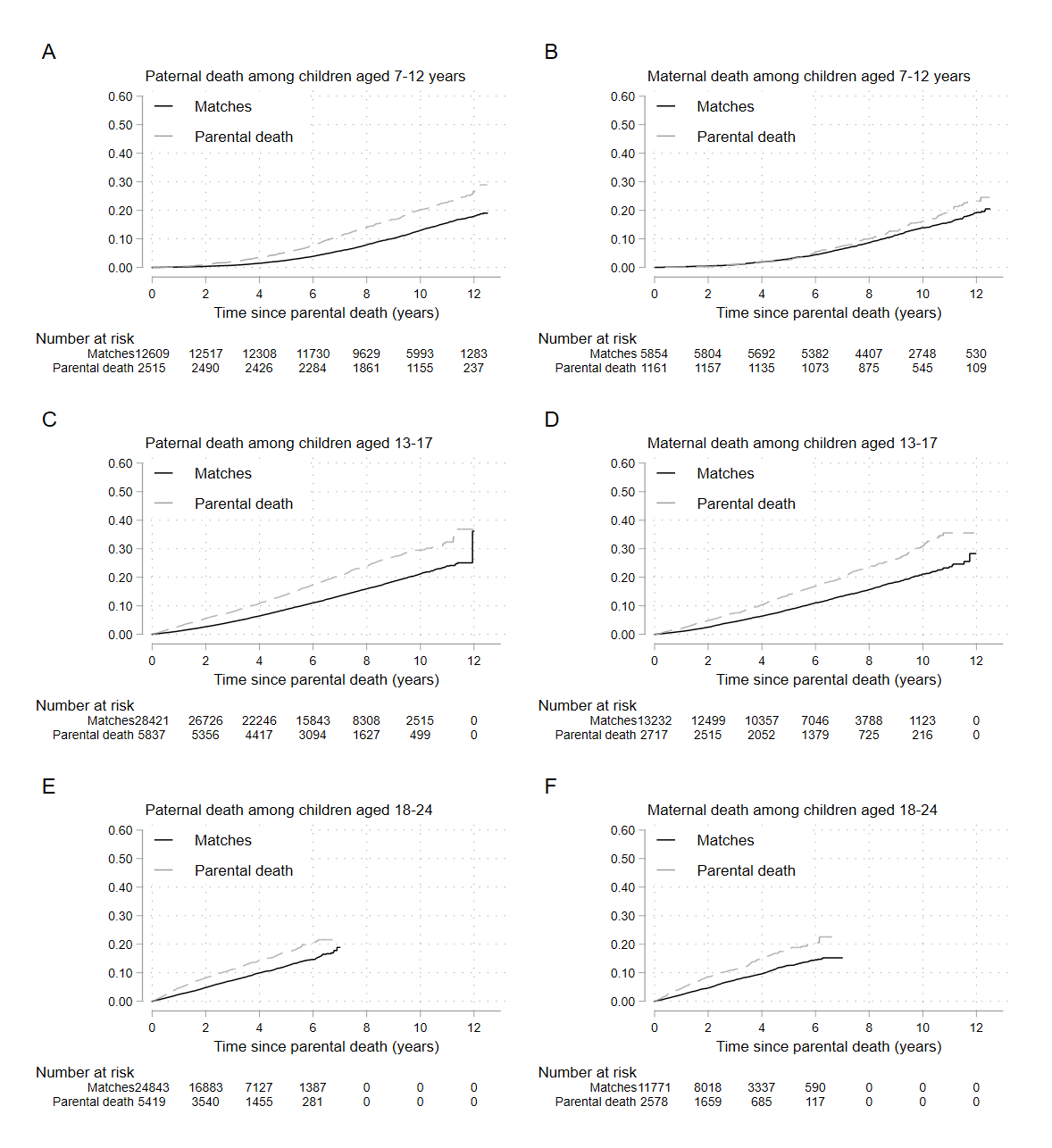


## Supplementary Figure 4a. Nelson-Aalen cumulative hazard functions of initiating antidepressant treatment after parental death, by age at parental death (A, C, and E: paternal death at age 7-12, 13-17, and 18-24 years, B, D, and F: maternal death at age 7-12, 13-17, and 18-24 years)

**
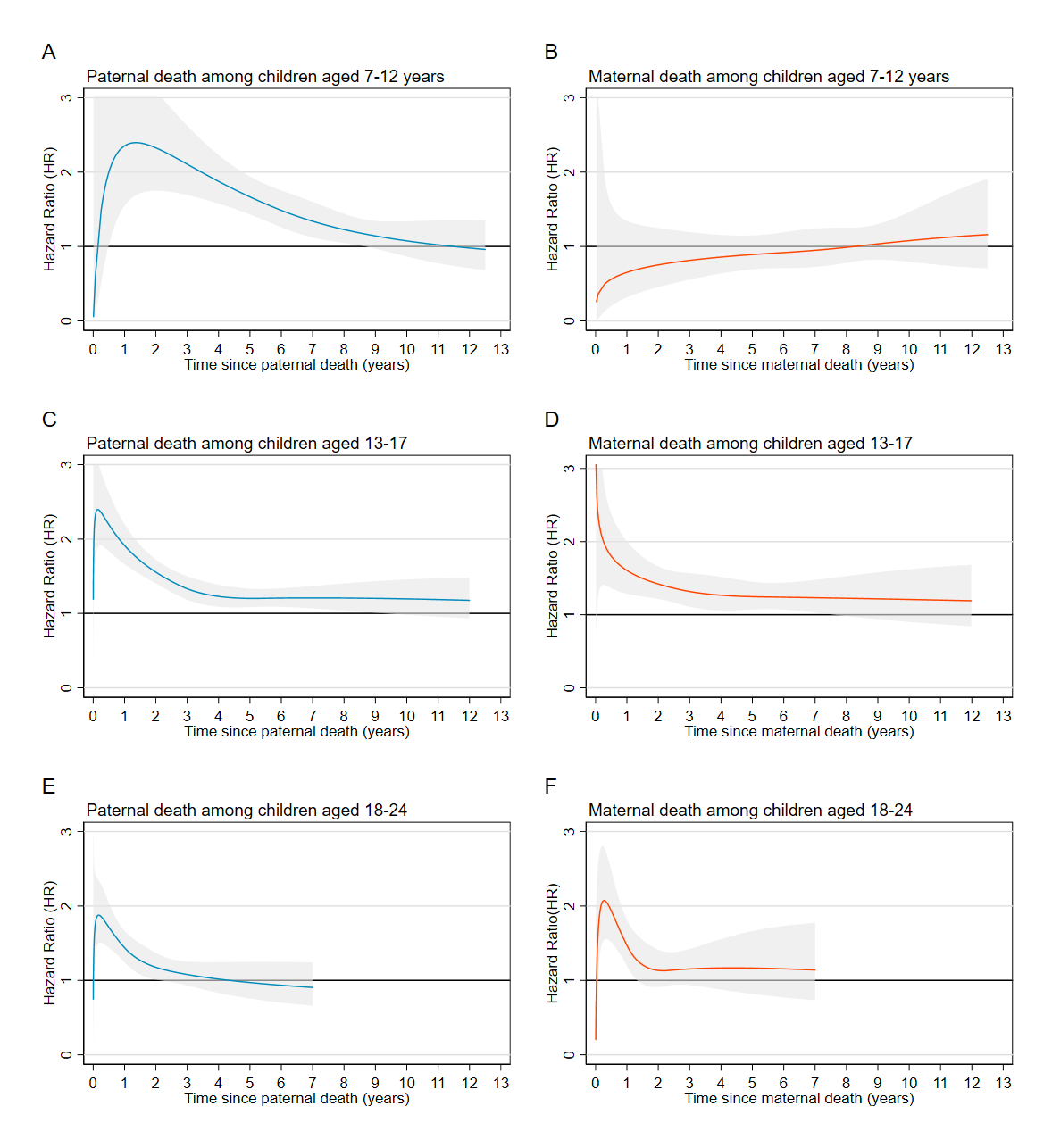
**

## Supplementary Figure 4b. Time-varying hazard ratio of initiating antidepressant treatment after parental death, by age at parental death (A, C, and E: paternal death at age 7-12, 13-17, and 18-24 years, B, D, and F: maternal death at age 7-12, 13-17, and 18-24 years)

Hazard ratio adjusted for sex, year of birth, birth order, maternal age and maternal residence county in the year of childbirth, maternal and paternal education, parental foreign-born status, and deceased parent having a psychiatric diagnosis before the death. Shaded areas indicate 95% confidence interval.

**
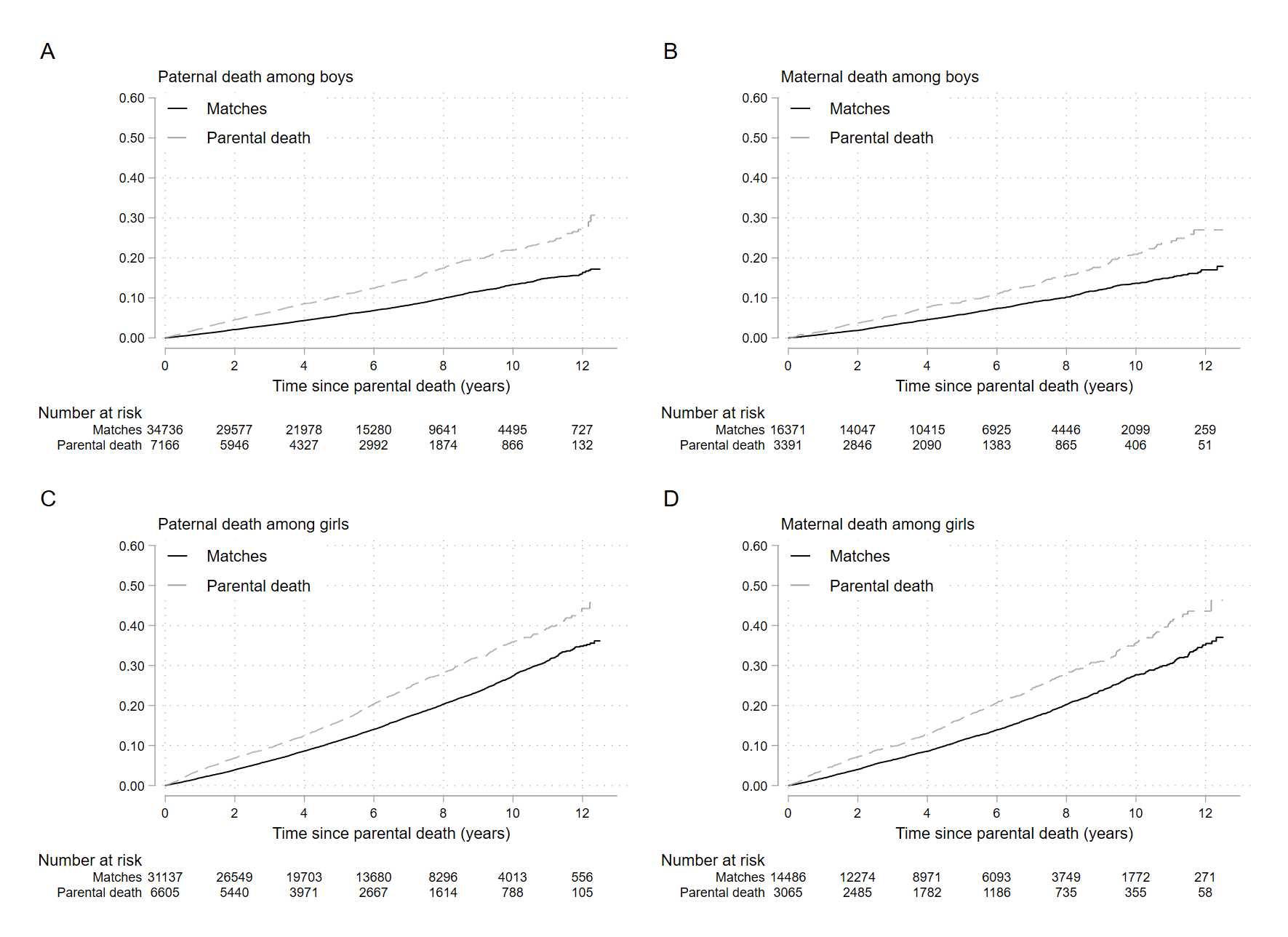
**

## Supplementary Figure 5a. Nelson-Aalen cumulative hazard functions of initiating antidepressant treatment after parental death, by sex of the child (A and C: paternal death among boys and girls, B and D: maternal death among boys and girls)

**
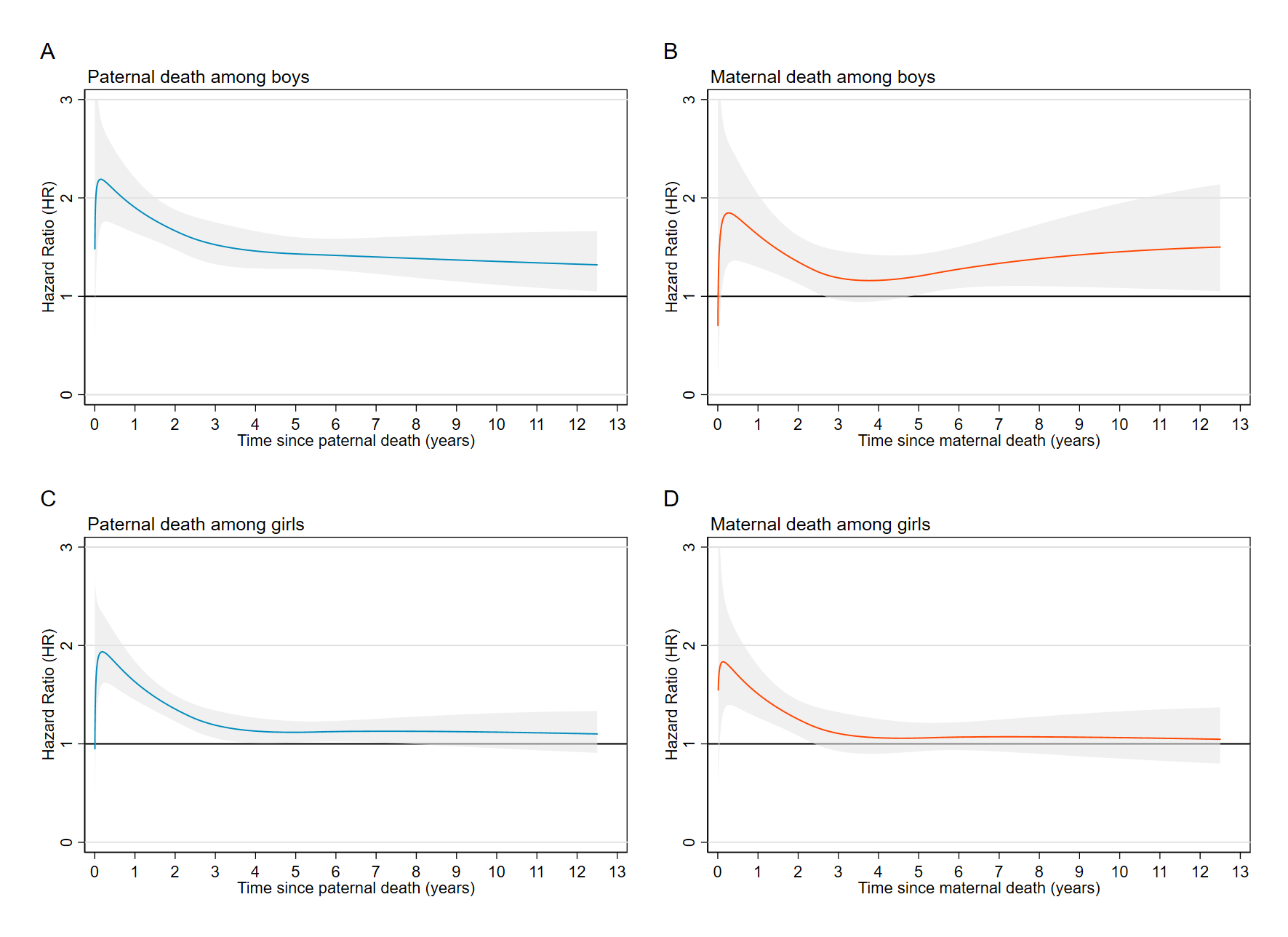
**

## Supplementary Figure 5b. Time-varying hazard ratio of initiating antidepressant treatment after parental death, by sex of the child (A and C: paternal death among boys and girls, B and D: maternal death among boys and girls)

Hazard ratio adjusted for year of birth, birth order, maternal age and maternal residence county in the year of childbirth, maternal and paternal education, parental foreign-born status, and deceased parent having a psychiatric diagnosis before the death. Shaded areas indicate 95% confidence interval.

**
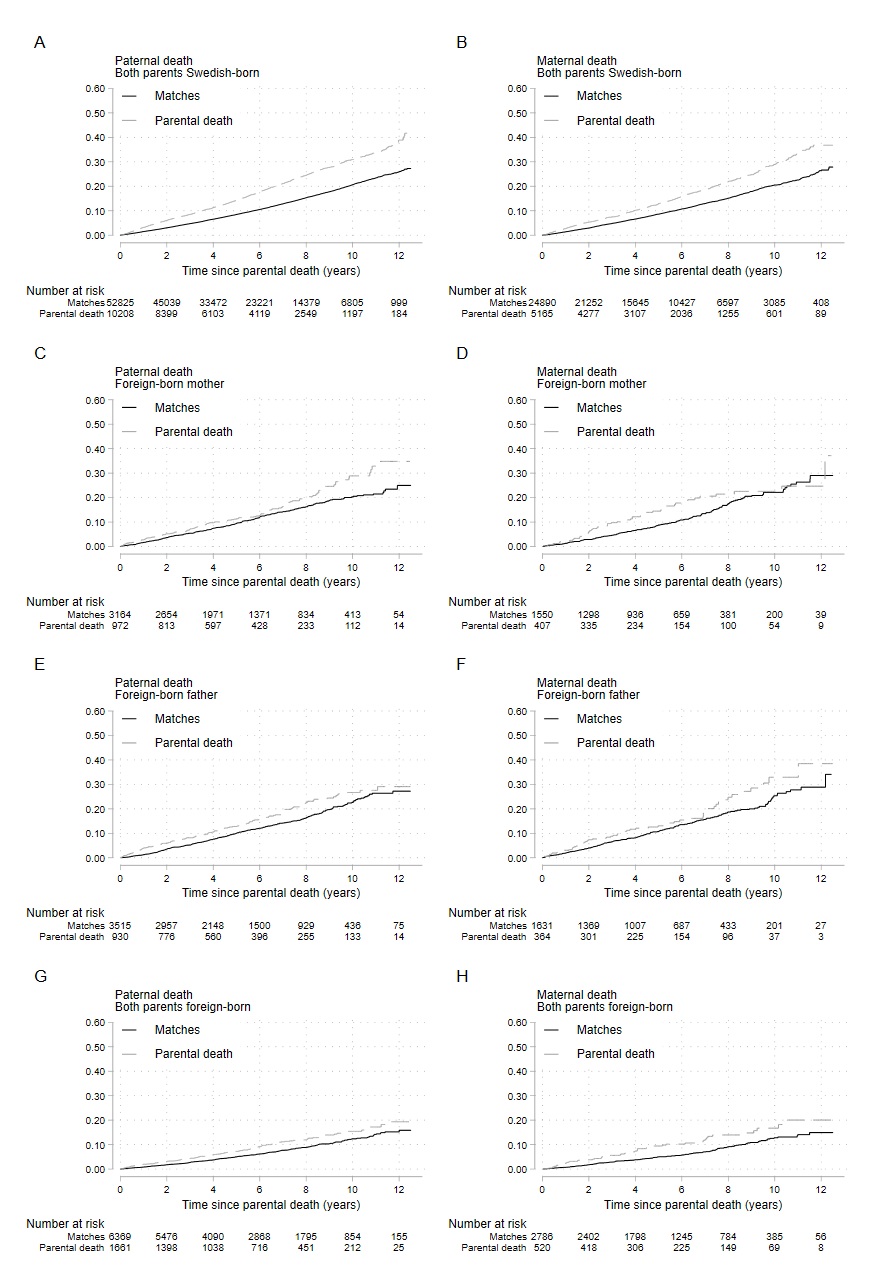
**

## Supplementary Figure 6a. Nelson-Aalen cumulative hazard functions of initiating antidepressant treatment after parental death, by parental foreign-born status (A and B: paternal and maternal death with both parents being Swedish-born, C and D: paternal and maternal death with a foreign-born mother and a Swedish-born father, E and F: paternal and maternal death with a foreign-born father and a Swedish-born mother, G and H: paternal and maternal death with both parents being foreign-born)

**
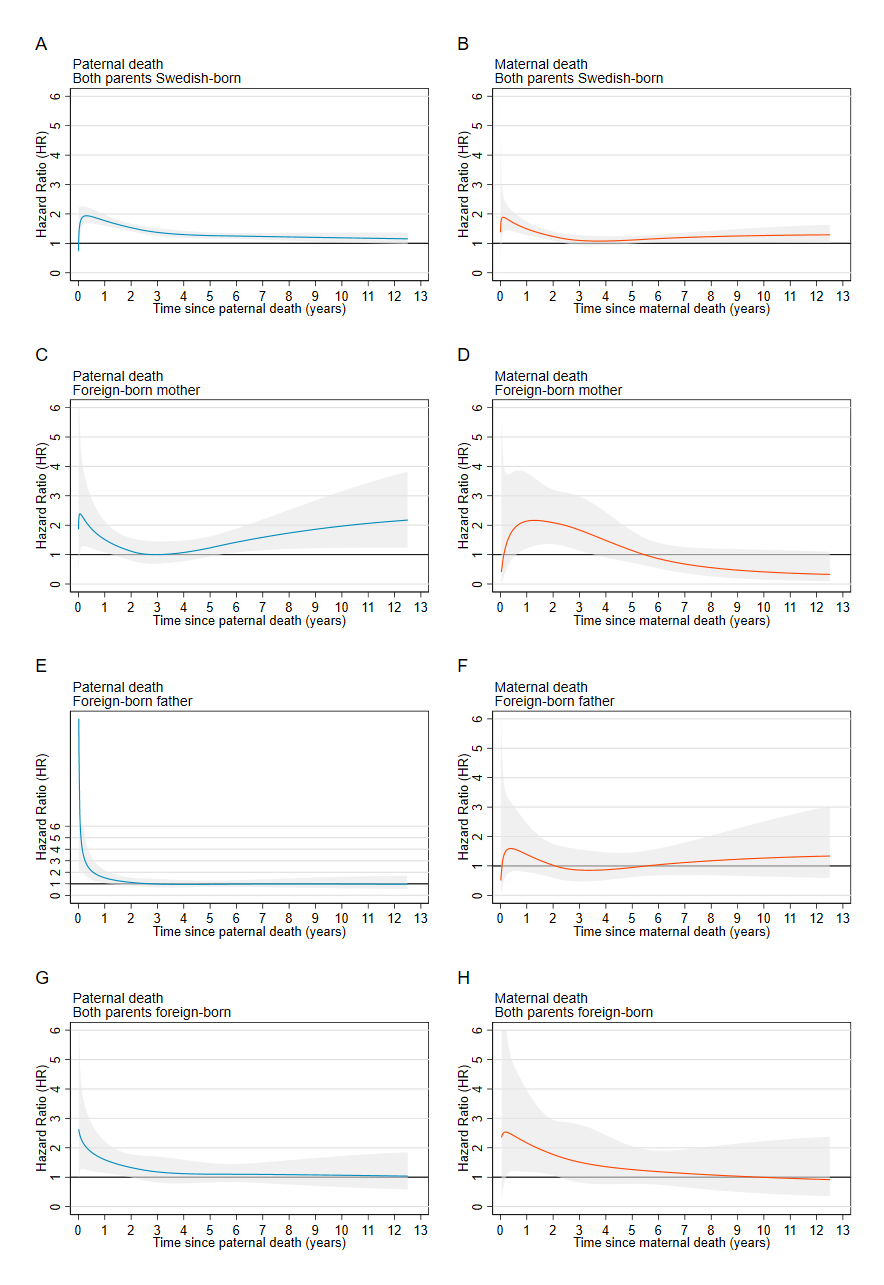
**

## Supplementary Figure 6b. Time-varying hazard ratio of initiating antidepressant treatment after parental death, by parental foreign-born status (A and B: paternal and maternal death with both parents being Swedish-born, C and D: paternal and maternal death with a foreign-born mother and a Swedish-born father, E and F: paternal and maternal death with a foreign-born father and a Swedish-born mother, G and H: paternal and maternal death with both parents being foreign-born)

Hazard ratio adjusted for sex, year of birth, birth order, maternal age and maternal residence county in the year of childbirth, maternal and paternal education, and deceased parent having a psychiatric diagnosis before the death. Shaded areas indicate 95% confidence interval.

**
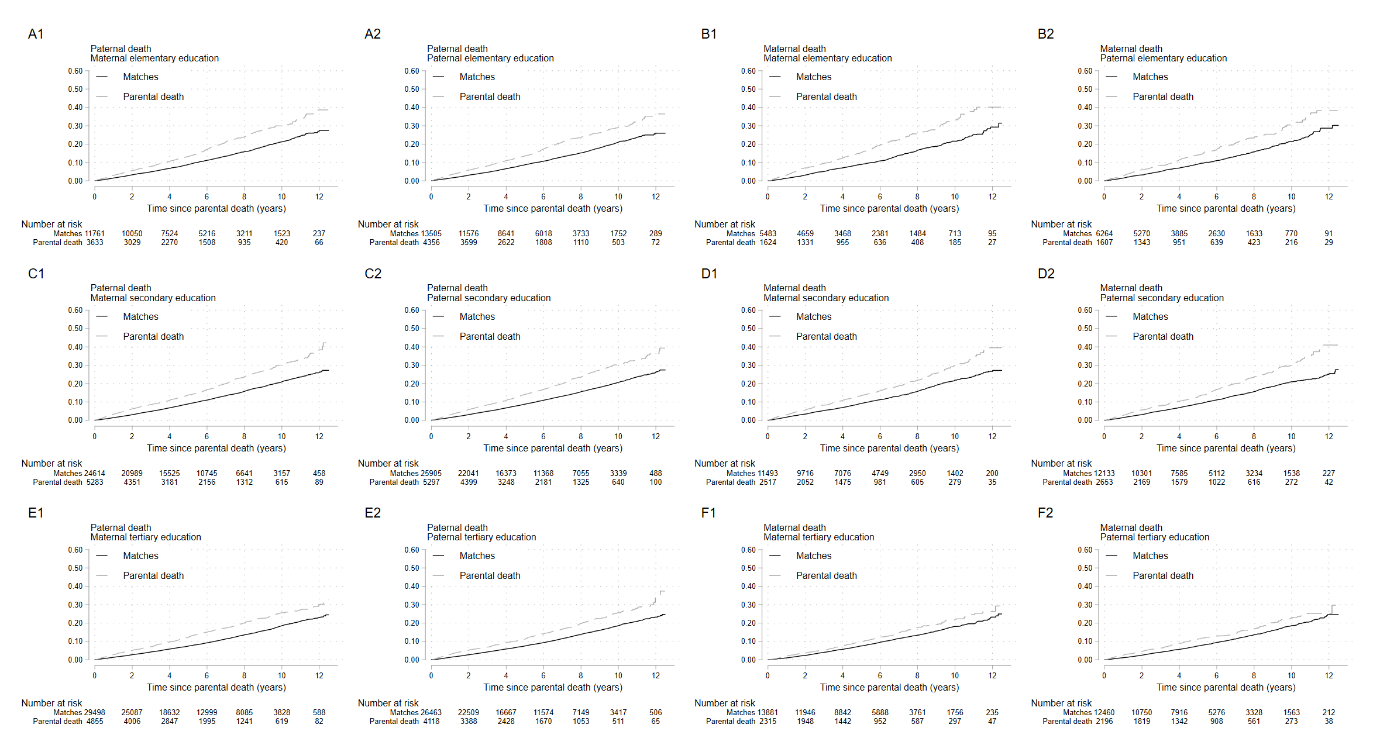
**

## Supplementary Figure 7a. Nelson-Aalen cumulative hazard functions of initiating antidepressant treatment after parental death, by maternal or paternal education (A1 and A2: paternal death with maternal or paternal elementary education, C1 and C2: paternal death with maternal or paternal secondary education, E1 and E2: paternal death with maternal or paternal tertiary education. Correspondingly B1 and B2, D1 and D2, and F1 and F2 show maternal death with maternal or paternal elementary, secondary, and tertiary education)

**
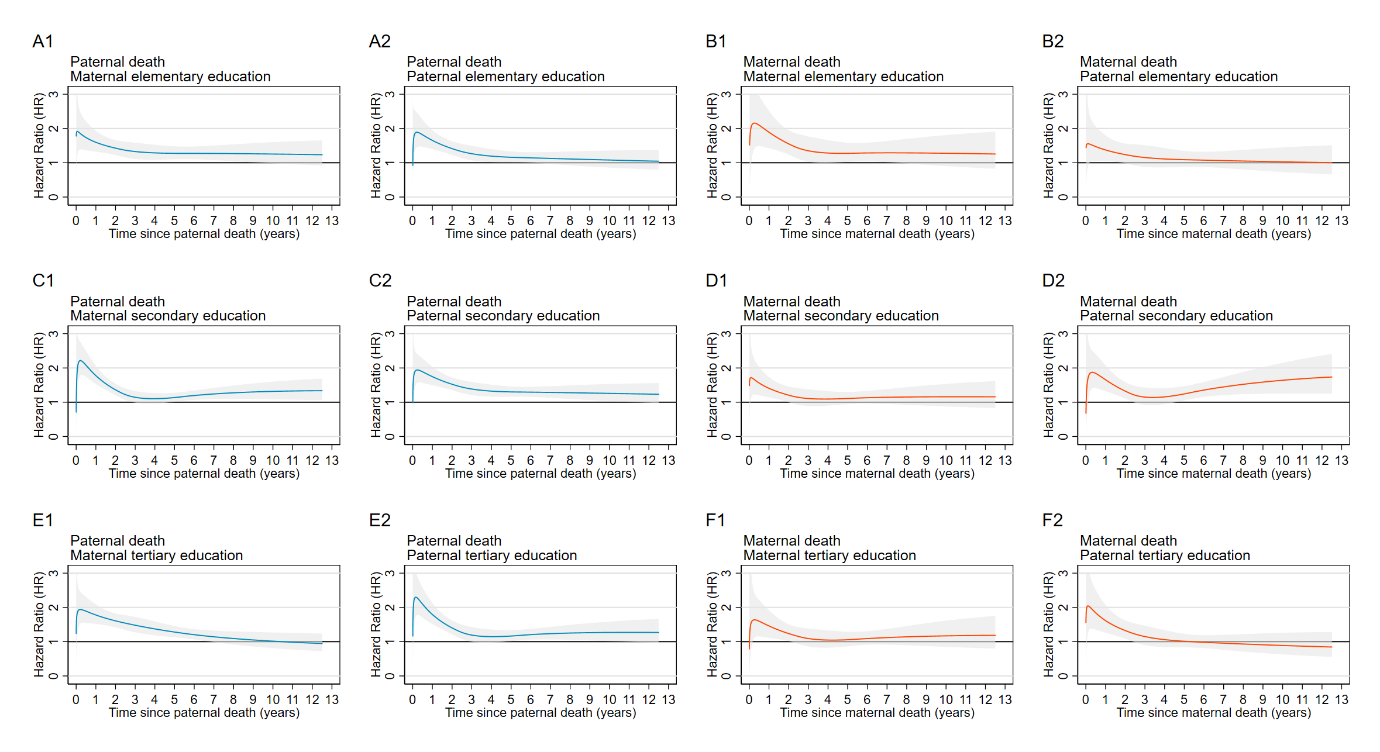
**

## Supplementary Figure 7b. Time-varying hazard ratio of initiating antidepressant treatment after parental death, by maternal or paternal education (A1 and A2: paternal death with maternal or paternal elementary education, C1 and C2: paternal death with maternal or paternal secondary education, E1 and E2: paternal death with maternal or paternal tertiary education. Correspondingly B1 and B2, D1 and D2, and F1 and F2 show maternal death with maternal or paternal elementary, secondary, and tertiary education)

Hazard ratio adjusted for sex, year of birth, birth order, maternal age and maternal residence county in the year of childbirth, paternal education, parental foreign-born status, and deceased parent having a psychiatric diagnosis before the death. Shaded areas indicate 95% confidence interval.


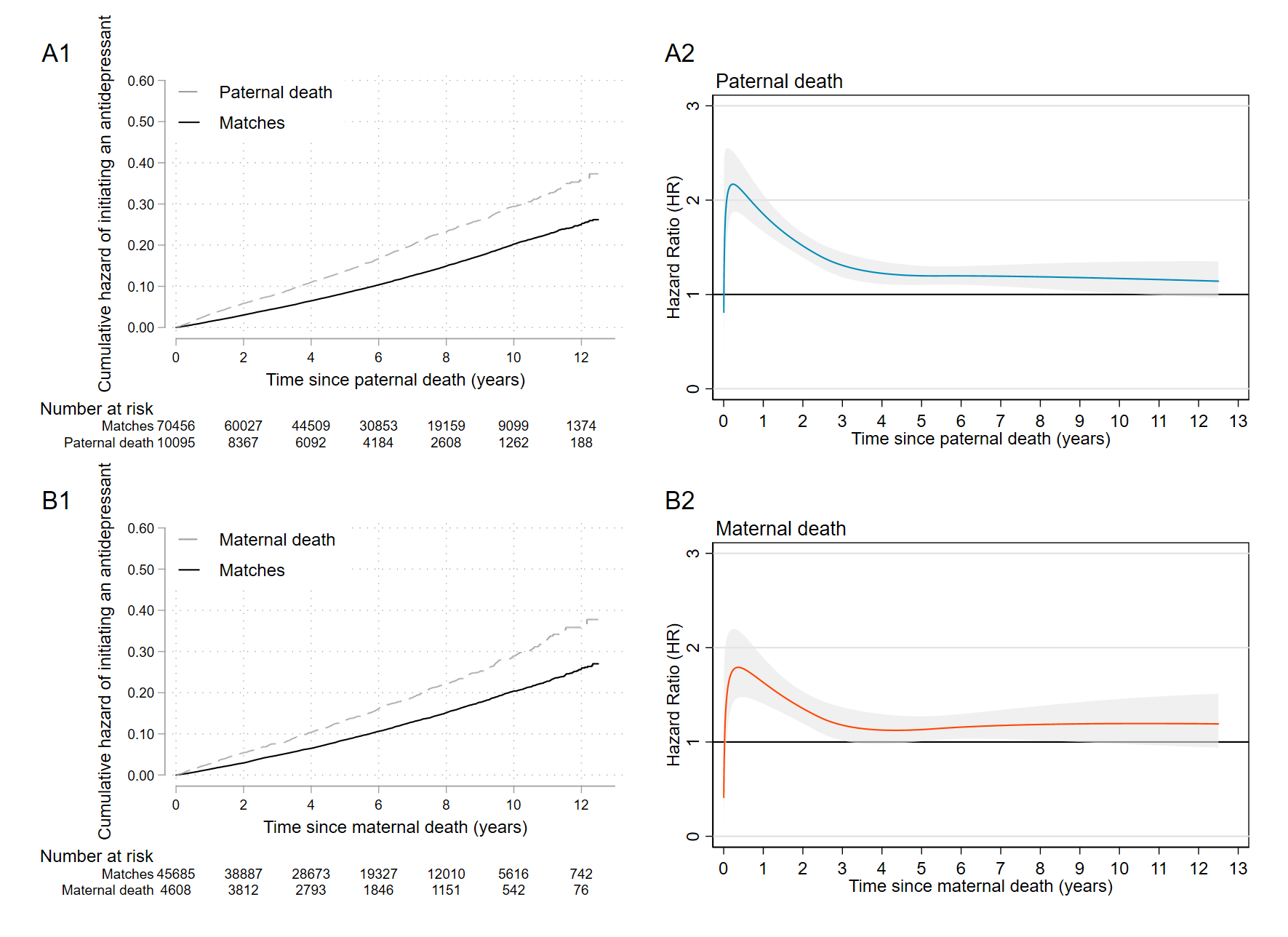


## Supplementary Figure 8. Nelson-Aalen cumulative hazard functions and time-varying hazard ratio of initiating antidepressant treatment after parental death, without sibling clustering (A1 and A2: paternal death, B1 and B2: maternal death)

Hazard ratio adjusted for sex, year of birth, birth order, maternal age and maternal residence county in the year of childbirth, maternal and paternal education, parental foreign-born status, and deceased parent having a psychiatric diagnosis before the death. Shaded areas indicate 95% confidence interval.

**
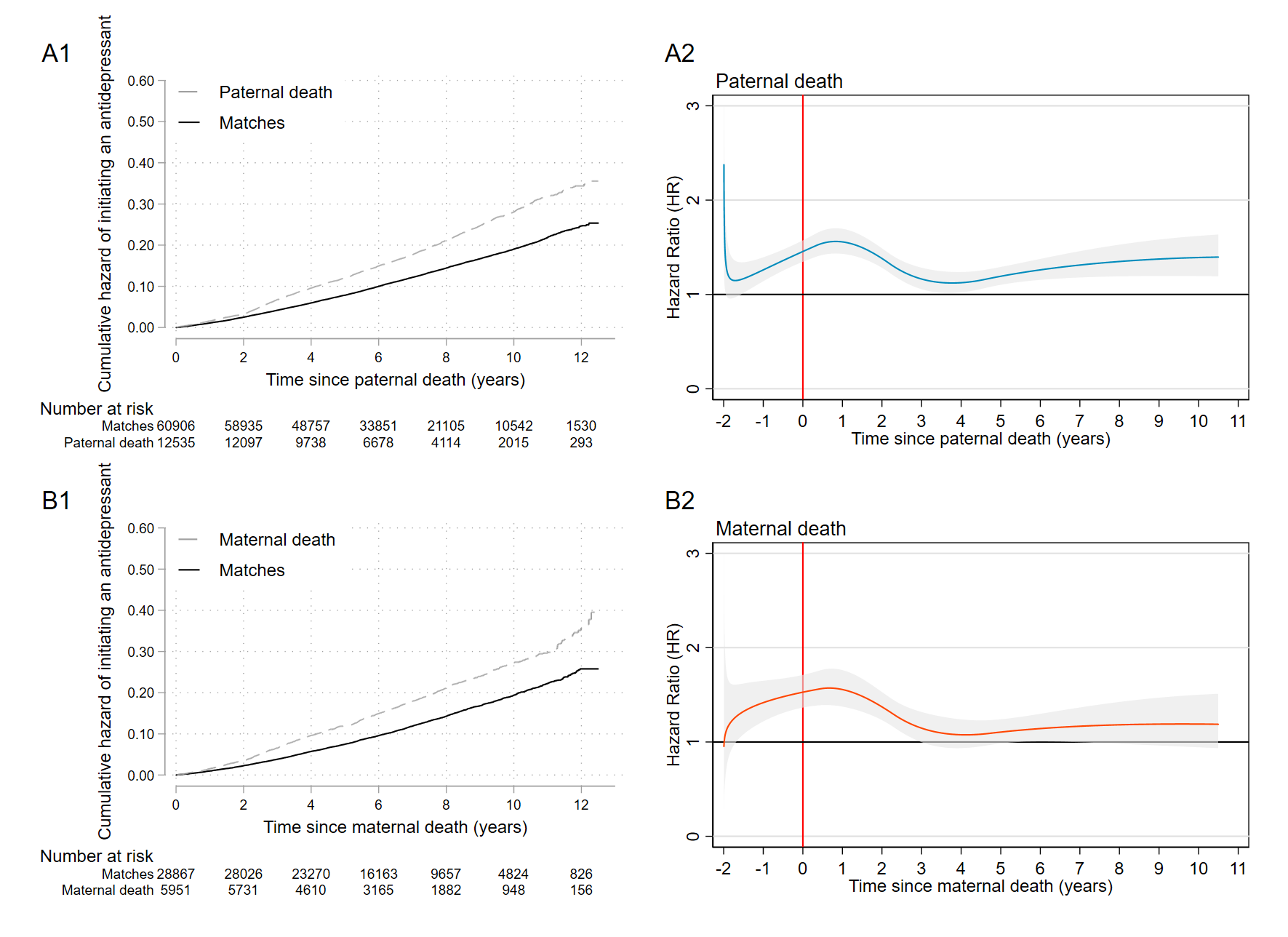
**

## Supplementary Figure 9. Nelson-Aalen cumulative hazard functions and time-varying hazard ratio of initiating antidepressant treatment after parental death, with start of follow-up from two years before the actual date of death (A1 and A2: paternal death, B1 and B2: maternal death)

Hazard ratio adjusted for sex, year of birth, birth order, maternal age and maternal residence county in the year of childbirth, maternal and paternal education, parental foreign-born status, and deceased parent having a psychiatric diagnosis before the death. Shaded areas indicate 95% confidence interval.
